# Adipose METTL14‐Elicited N^6^‐Methyladenosine Promotes Obesity, Insulin Resistance, and NAFLD Through Suppressing β Adrenergic Signaling and Lipolysis

**DOI:** 10.1002/advs.202301645

**Published:** 2023-08-01

**Authors:** Qianqian Kang, Xiaorong Zhu, Decheng Ren, Alexander Ky, Ormond A. MacDougald, Robert W. O'Rourke, Liangyou Rui

**Affiliations:** ^1^ Department of Molecular and Integrative Physiology University of Michigan Medical School Ann Arbor MI 48109 USA; ^2^ Elizabeth Weiser Caswell Diabetes Institute University of Michigan Ann Arbor MI 48109 USA; ^3^ Department of Endocrinology Beijing Tongren Hospital Capital Medical University Beijing Diabetes Institute Beijing 100730 China; ^4^ Department of Medicine University of Chicago Chicago IL 60637 USA; ^5^ Department of Surgery University of Michigan Medical School Ann Arbor MI 48109 USA; ^6^ Department of Internal Medicine University of Michigan Medical School Ann Arbor MI 48109 USA; ^7^ Department of Surgery Veterans Affairs Ann Arbor Healthcare System An Arbor MI 48105 USA

**Keywords:** β‐adrenergic signaling, adipose tissue, diabetes, lipolysis, m6A, obesity, RNA modifications

## Abstract

White adipose tissue (WAT) lipolysis releases free fatty acids as a key energy substance to support metabolism in fasting, cold exposure, and exercise. Atgl, in concert with Cgi‐58, catalyzes the first lipolytic reaction. The sympathetic nervous system (SNS) stimulates lipolysis via neurotransmitter norepinephrine that activates adipocyte β adrenergic receptors (Adrb1‐3). In obesity, adipose Adrb signaling and lipolysis are impaired, contributing to pathogenic WAT expansion; however, the underling mechanism remains poorly understood. Recent studies highlight importance of N^6^‐methyladenosine (m6A)‐based RNA modification in health and disease. METTL14 heterodimerizes with METTL3 to form an RNA methyltransferase complex that installs m6A in transcripts. Here, this work shows that adipose Mettl3 and Mettl14 are influenced by fasting, refeeding, and insulin, and are upregulated in high fat diet (HFD) induced obesity. Adipose *Adrb2*, *Adrb3*, *Atgl*, and *Cgi‐58* transcript m6A contents are elevated in obesity. Mettl14 ablation decreases these transcripts’ m6A contents and increases their translations and protein levels in adipocytes, thereby increasing Adrb signaling and lipolysis. Mice with adipocyte‐specific deletion of *Mettl14* are resistant to HFD‐induced obesity, insulin resistance, glucose intolerance, and nonalcoholic fatty liver disease (NAFLD). These results unravel a METTL14/m6A/translation pathway governing Adrb signaling and lipolysis. METTL14/m6A‐based epitranscriptomic reprogramming impairs adipose Adrb signaling and lipolysis, promoting obesity, NAFLD, and metabolic disease.

## Graphical abstract

Mettl3/Mettl14 complex installs N6‐methyladenosine (m6A) in RNA to shape RNA fate. In adipocytes, Mettl3 and Mettl14 deposit m6A in β adrenergic signaling and lipolytic transcripts to decrease their translations and protein levels, thereby suppressing β adrenergic signaling and lipolysis. Adipose Mettl3 and Mettl14 are upregulated in obesity. Adipocyte‐specific deletion of Mettl14 protects against diet‐induced obesity, NAFLD, and metabolic disease through increasing adipose β adrenergic signaling and lipolysis.

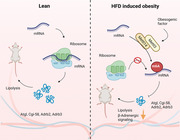

## 1 Introduction

Excessive expansion of white adipose tissue (WAT), a hallmark of obesity, is a risk factor for metabolic disease, and the underlying mechanism is complex and poorly understood. Adipocyte‐specific ablation of adipose triglyceride lipase (ATGL), which catalyzes the first reaction of lipolysis and is downregulated in obesity, results in excessive WAT expansion.^[^ ^1^ ^]^ Conversely, adipocyte‐specific overexpression of ATGL, or ablation of ATGL suppressor G0S2, protects against obesity and obesity‐associated metabolic disorders.^[^ ^2^ ^]^ These observations raise the possibility that ATGL downregulation and lipolysis suppression may play an important role in obesity development. Indeed, in both humans and mice, adipose lipolysis declines in obesity and aging (often associated with obesity).^[^ ^1^, ^3^ ^]^ Adipose lipolysis is tightly regulated by the sympathetic nervous system (SNS) and insulin. The SNS stimulates lipolysis via neural transmitter norepinephrine that binds to β adrenergic receptor 1–3 (Adrb1‐3) to activate the cAMP/protein kinase A (PKA) pathway in adipocytes.^[^ ^4^ ^]^ PKA phosphorylates ATGL and hormone‐sensitive lipase (HSL) and increases their lipase activities.^[^ ^4^, ^5^ ^]^ PKA also phosphorylates comparative gene identification 58 (CGI‐58, also known as ABHD5) and increases the ability of CGI‐58 to stimulate ATGL.^[^ ^6^ ^]^ Insulin suppresses lipolysis by activating PDE3B to counteract β adrenergic signaling.^[^ ^7^ ^]^ Additionally, insulin stimulates expression of Snail1 that epigenetically suppresses ATGL expression and lipolysis in WAT.^[^ ^1a^ ^]^ Of note, adipose β adrenergic signaling is impaired in obesity, leading to catecholamine resistance.^[^ ^8^ ^]^ Reversing catecholamine resistance, by either genetic or pharmacological intervention, mitigates high fat diet (HFD) induced obesity and insulin resistance in mice.^[^ ^7^, ^8^, ^9^ ^]^ Thus, impaired adipose β adrenergic signaling contributes to lipolysis inhibition and WAT expansion in obesity.

Recent studies highlight the importance of RNA modifications in health and disease.^[^ ^10^ ^]^ N^6^‐methyladenosine (m6A) is the predominant form of mRNA modifications.^[^ ^11^ ^]^ METTL14 (structural subunit) binds to METTL3 (catalytic subunit) to assemble a functional m6A methyltransferase, referred to as the m6A writer that deposits m6A on mRNA as well as noncoding RNA.^[^ ^12^ ^]^ The m6A motifs are recognized and bound by RNA‐binding proteins, referred to as m6A readers which in turn regulate the metabolism and fate of target RNAs.^[^ ^10^, ^13^ ^]^ Global deletion of either *Mettl14* or *Mettl3* results in embryonic death in mice,^[^ ^14^ ^]^ indicating that m6A modification is essential for survival. Pancreatic β cell‐specific deletion of *Mettl14* impairs insulin secretion and promotes diabetes.^[^ ^15^ ^]^ Liver‐specific deletion of *Mettl3* attenuates HFD‐induced insulin resistance and liver steatosis.^[^ ^16^ ^]^ These observations indicate that the Mettl14/Mettl3/m6A system in metabolic tissues plays an important role in the maintenance of metabolic homeostasis. However, the Mettl14/m6A system has not been explored in WAT and lipolysis. In this study, we identify *Atgl*, *Cgi‐58*, *Adrb2*, and *Adrb3* transcripts as previously unrecognized targets of Mettl14. Mettl14‐elicited m6A modification suppresses their translations, resulting in catecholamine resistance and lipolysis inhibition in WAT. Adipocyte‐specific deletion of *Mettl14* increases adipose lipolysis and protects against HFD‐induced obesity, insulin resistance, and nonalcoholic fatty liver disease (NAFLD). These results unveil a Mettl14/m6A/β adrenergic signaling circuit and a Mettl14/m6A/lipolysis axis that play a pivotal role in WAT expansion and obesity progression.

## 2 Results

### 2.1 Mettl3/Mettl14/m6A Pathway is Stimulated in WAT by Refeeding, Insulin, and Obesogenic Factors

**Figure 1:**
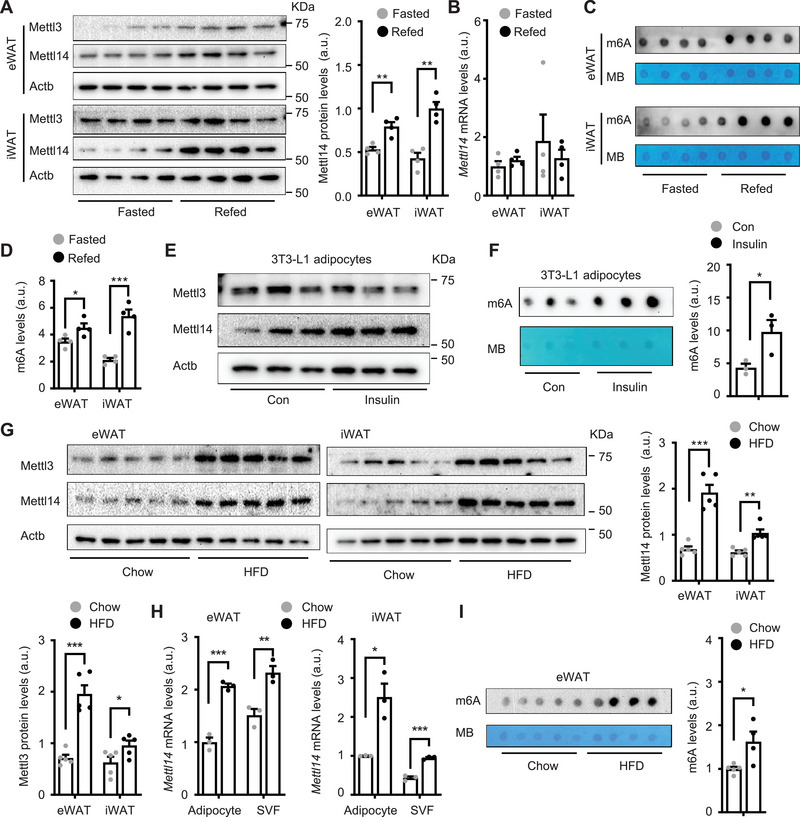
Mettl3/Mettl14/m6A‐based RNA modification is upregulated in fat by insulin and feeding and in obesity. A–D) C57BL/6J male mice (8 weeks old) were fasted overnight and then refed for 3 h. White adipose tissue (WAT) was harvested. A) epididymal WAT (eWAT) and inguinal WAT (iWAT) extracts were immunoblotted with the indicated antibodies. Mettl14 levels were normalized to Actb levels (*n* = 4 mice per group). a.u.: arbitrary unit. B) Mettl14 mRNA levels were measured by qPCR and normalized to 36B4 levels (*n* = 4 mice per group). C,D) Total m6A and total RNA levels were measured by dot blot assays and methylene blue (MB) staining. M6A levels were normalized to total RNA levels (*n* = 4 mice per group). E,F) 3T3‐L1 cells were differentiated into adipocytes. E) Cell extracts were immunoblotted with the indicated antibodies. F) m6A levels were measured by dot blot assays and normalized to total RNA levels (*n* = 3 repeats per group). G–I) C57BL/6J male mice (8 weeks old) were fed a high fat diet (HFD) for 12 weeks. G) iWAT and eWAT extracts were immunoblotted with the indicated antibodies (overnight fasting). Mettl3 and Mettl14 levels were quantified and normalized to Actb levels (*n* = 5 mice per group). H) Adipocytes and stromal vascular fraction (SVF) cells were purified from iWAT and eWAT. Mettl14 mRNA abundance was measured by qPCR and normalized to 36B4 levels (*n* = 3 mice per group). I) m6A levels in eWAT were measured by dot blot assays and normalized to total RNA levels. Chow: *n* = 5, HFD: *n* = 4. Data are presented as mean ± SEM. *p < 0.05, **p < 0.01, ***p < 0.001, Student's *t* test.

To examine the influences of nutrients and metabolic hormones on adipose m6A modification of RNA transcripts, C57BL/6J male mice were fasted overnight and refed for 3 h. Inguinal (iWAT) and epididymal WAT (eWAT) were dissected to measure the protein and mRNA levels of Mettl3 and Mettl14 by immunoblotting and qPCR, respectively. Refeeding increased protein, but not mRNA, levels of Mettl14 in both iWAT and eWAT (**Figure** **1**A,B). Refeeding increased Mettl3 levels in eWAT but not iWAT (Figure 1A and Figure S1A, Supporting Information). Thus, Mettl3 and Mettl14 may be regulated by different mechanisms in iWAT and eWAT. We next measured adipose m6A levels using m6A dot blot assays. Refeeding significantly increased m6A levels in iWAT and eWAT (Figure 1C,D). Considering that refeeding stimulates insulin secretion, we tested if insulin increases Mettl3, Mettl14, and m6A levels. 3T3‐L1 cells were differentiated into adipocytes and stimulated with insulin in vitro. As expected, insulin stimulated phosphorylation of Akt and Gsk3β (Figure S1B, Supporting Information). Insulin stimulation increased Mettl14 and m6A levels (Figure 1E,F). Given that obesity is associated with hyperinsulinemia, we tested if adipose Mettl14 and m6A are elevated in obesity. We placed C57BL/6J male mice on a HFD for 12 weeks (chow: body weight 26.84 ± 1.31 g, *n* = 5, HFD: 51.48 g ± 0.70 g, *n* = 6, *p* < 0.001) and harvested WAT after overnight fasting. Mettl3 and Mettl14 protein levels in both iWAT and eWAT were substantially higher in HFD‐fed than in chow‐fed mice (Figure 1G). After HFD feeding for 16 weeks when fasted levels of Mettl3 and Mettl14 are markedly elevated, refeeding slightly increased Mettl14 levels in iWAT and was unable to increase Mettl3 and Mettl14 levels in eWAT (Figure S1C, Supporting Information). To further examine impact of obesogenic factors on the Mettl14/m6A system in adipocytes, we placed mice on HFD for 12 weeks and purified primary adipocytes and stromal vascular fraction (SVF) cells from iWAT and eWAT. HFD feeding increased *Mettl14* mRNA abundance in both adipocytes and SVF cells from iWAT and eWAT (Figure 1H). Consistently, adipocyte m6A levels were significantly higher in HFD‐fed than in chow‐fed mice (Figure 1I). These results demonstrate that refeeding, insulin, and obesogenic factors increase adipose Mettl14 expression and m6A levels.

### 2.2 Adipocyte‐Specific Deletion of Mettl14 Protects Against HFD‐Induced Obesity

**Figure 2:**
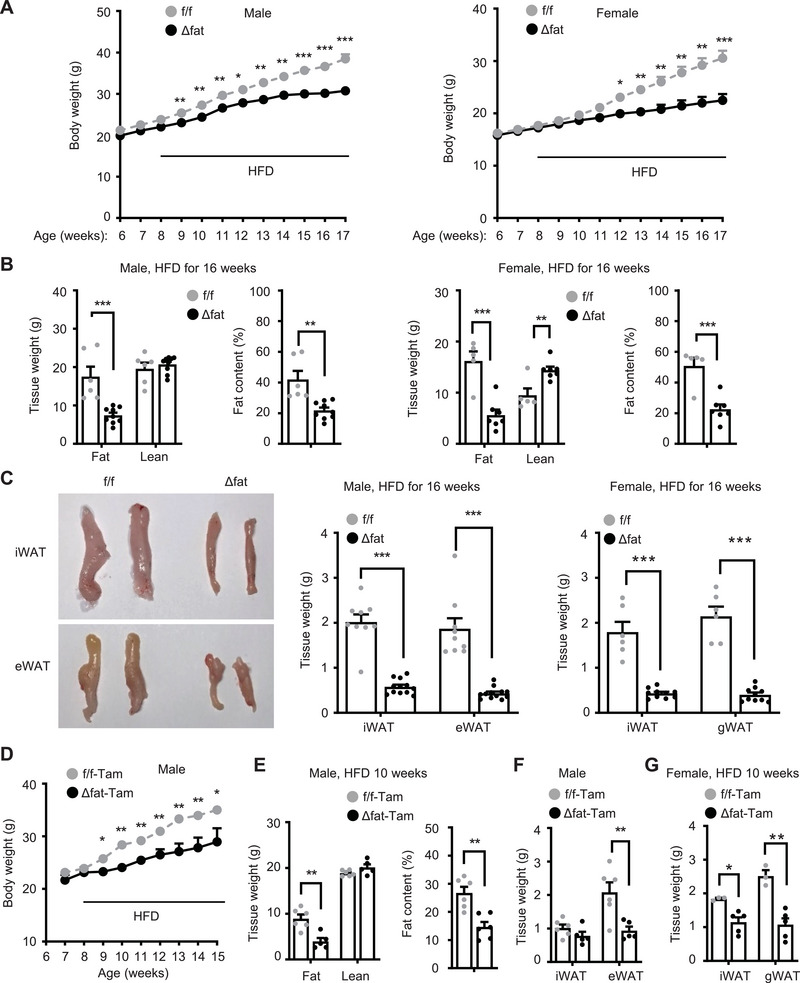
Adipocyte‐specific deletion of Mettl14 protects against diet‐induced obesity. Male and female mice (8 weeks) were fed a high fat diet (HFD) for 10 weeks. A) Grown curves. Male Mettl14f/f: *n* = 9, male Mettl14Δfat: *n* = 12, female Mettl14f/f: *n* = 6, female Mettl14Δfat: *n* = 10. B) Fat content and lean mass were measured by pDexa (HFD for 10 weeks). Male Mettl14f/f: *n* = 6, male Mettl14Δfat: *n* = 9, female Mettl14f/f: *n* = 5, female Mettl14Δfat: *n* = 7. C) White adipose tissue (WAT) weights on HFD for 16 weeks. Male Mettl14f/f: *n* = 9, male Mettl14Δfat: *n* = 12, female Mettl14f/f: *n* = 6, female Mettl14Δfat: *n* = 10. D–G) Mice (7 weeks) were injected with tamoxifen and fed a HFD. D) Growth curves. f/f‐Tam: *n* = 6, Δfat‐Tam: *n* = 5. E) Fat content and lean mass on HFD for 10 weeks. f/f‐Tam: *n* = 6, Δfat‐Tam: *n* = 5. F,G) WAT weights on HFD for 10 weeks. Male Mettl14f/f: *n* = 6, male Mettl14Δfat: *n* = 5, female Mettl14f/f: *n* = 3, female Mettl14Δfat: *n* = 5. Data are presented as mean ± SEM. *p < 0.05, **p < 0.01, ***p < 0.001, Student's *t* test (B,C,E–G) and ANOVA (A,D).

To explore adipose m6A function, we generated adipocyte‐specific *Mettl14* knockout (*Mettl14^Δfat^*) mice by crossing *Mettl14^f/f^* mice with *adiponectin‐cre* mice. *Mettl14^f/f^* mice were described before.^[^ ^15^, ^17^ ^]^ *Mettl14* mRNA levels in iWAT and eWAT were markedly lower in *Mettl14^Δfat^* than in *Mettl14^f/f^* mice (Figure S1D, Supporting Information). Adipocyte m6A levels were also lower in *Mettl14^Δfat^* mice relative to *Mettl14^f/f^* mice (Figure S1E, Supporting Information). Mettl14 protein levels were decreased in eWAT, iWAT, and brown adipose tissue (BAT), but not in the skeletal muscle, heart, liver, spleen, lung, and kidneys, in *Mettl14^Δfat^* mice (Figure S1F, Supporting Information). The residual Mettl14 in WAT of *Mettl14^Δfat^* mice might come from nonadipocyte cells. Body weight, glucose tolerance tests (GTT), and insulin tolerance testes (ITT) were comparable between *Mettl14^Δfat^* and *Mettl14^f/f^* mice on a chow diet (Figure S1G,H, Supporting Information). Next, we placed *Mettl14^f/f^* and *Mettl14^Δfat^* littermates on a HFD for 16 weeks to increases adipose Mettl14 expression in *Mettl14^f/f^* (but not *Mettl14^Δfat^*) mice. Body weight was markedly lower in *Mettl14^Δfat^* than in *Mettl14^f/f^* littermates, in both males and females (**Figure** **2**A and Figure S2A, Supporting Information). Body weight difference between *Mettl14^Δfat^* and *Mettl14^f/f^* mice became larger progressively after HFD. Whole body fat content was significantly lower in *Mettl14^Δfat^* mice relative to *Mettl14^f/f^* mice (both male and female), while lean mass was comparable between the two groups (Figure 2B). Weights of iWAT (male and female), eWAT (male), and gonadal WAT (gWAT, female) were substantially lower in *Mettl14^Δfat^* than in *Mettl14^f/f^* mice (Figure 2C). Adipocyte size was smaller in *Mettl14^Δfat^* than in *Mettl14^f/f^* mice (Figure S2B, Supporting Information). To assess the influence of adipose development on the phenotypes, we generated tamoxifen‐induced, adult‐onset, and adipocyte‐specific *Mettl14* knockout mice (*Mettl14^Δfat‐Tam^*) by crossing *Mettl14^f/f^* mice with *adiponectin‐CreERT* mice. *Mettl14^Δfat‐Tam^* male mice (7 weeks old) were injected with tamoxifen to delete adipose *Mettl14*, and *Mettl14^f/f^* littermates were injected with tamoxifen as control. One week later, the mice were fed a HFD for additional 7 weeks. *Mettl14^Δfat‐Tam^* mice were resistant to diet‐induced obesity (Figure 2D). Fat content and weights of eWAT were significantly lower in *Mettl14^Δfat‐Tam^* than in *Mettl14^f/f‐Tam^* mice (Figure 2E,F). Likewise, *Mettl14^Δfat‐Tam^* females were also resistant to HFD‐induced obesity (*Mettl14^Δfat‐Tam^*: 26.36 ± 0.84 g, *n* = 5, *Mettl14^f/f‐Tam^*: 30.4 ± 1.0 g, *n* = 3, p = 0.023, HFD for 7 weeks). WAT weights were substantially lower in *Mettl14^Δfat‐Tam^* mice relative to *Mettl14^f/f‐Tam^* females on HFD for 10 weeks (Figure 2G). Collectively, these results demonstrate, for the first time, that adipose *Mettl14*/m6A pathway is required for diet‐induced obesity.

### 2.3 Adipocyte‐Specific Deletion of Mettl14 Attenuates HFD‐Induced Insulin Resistance, Glucose Intolerance, and NAFLD

**Figure 3:**
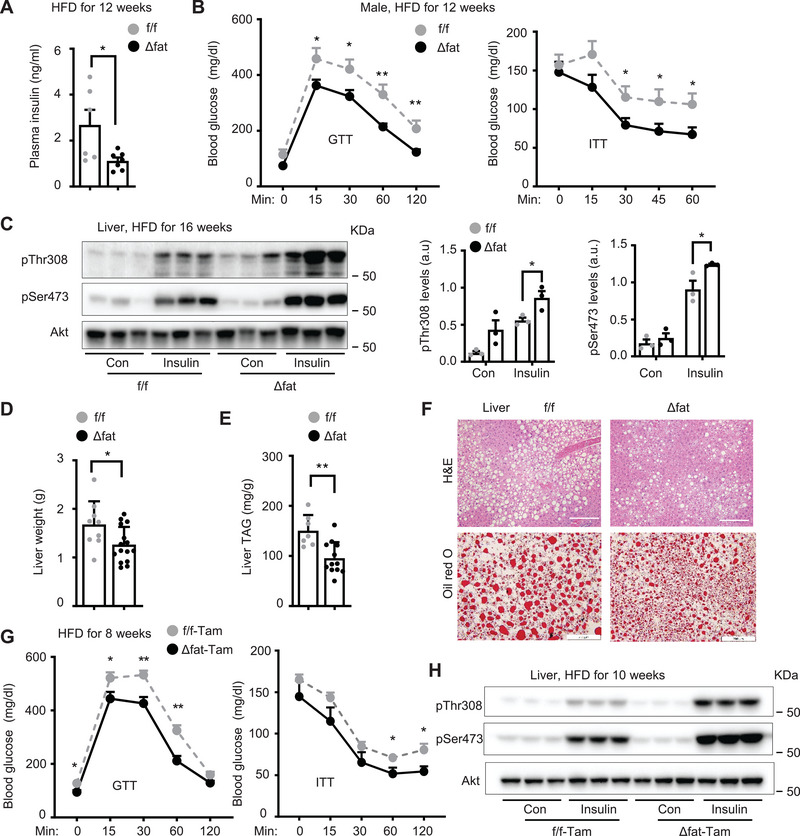
Mettl14Δfat mice are resistant to high fat diet (HFD) induced insulin resistance, glucose intolerance, and nonalcoholic fatty liver disease (NAFLD). Male mice were fed a HFD at 8 weeks of age. A) Overnight fasted blood insulin on HFD for 12 weeks. Mettl14f/f: *n* = 6, Mettl14Δfat: *n* = 7. B) Glucose tolerance tests (GTT) on HFD for 12 weeks (Mettl14f/f: *n* = 6, Mettl14Δfat: *n* = 8) and insulin tolerance testes (ITT) on HFD for 12 weeks (Mettl14f/f: *n* = 8, Mettl14Δfat: *n* = 14). C) Mice (HFD for 16 weeks) were fasted overnight and injected with insulin (0.75 unit kg^−1^ body weight) for 5 min. Liver extracts were immunoblotted with anti‐phospho‐Akt (pThr308, pSer473). Akt phosphorylation was normalized to total Akt levels (*n* = 3 mice per group). a.u.: arbitrary unit. D) Liver weight on HFD for 16 weeks. Mettl14f/f: *n* = 9, Mettl14Δfat: *n* = 15. E) Liver triacylglycerol (TAG) on HFD for 16 weeks (normalized to body weight). Mettl14f/f: *n* = 7, Mettl14Δfat: *n* = 12. F) H&E and Oil red O staining of liver sections on HFD for 16 weeks (*n* = 3 mice per group). Scale bar: 200 µm. G‐H) Mettl14f/f‐Tam and Mettl14Δfat‐Tam male mice (7 weeks) were injected with tamoxifen. One week later, they were fed a HFD for 10 weeks. G) GTT and ITT. Mettl14f/f‐Tam: *n* = 6, Mettl14Δfat‐Tam: *n* = 5. H) Mice were fasted overnight and injected with insulin (0.75 unit kg^−1^ body weight) for 5 min. Liver extracts were immunoblotted with the indicated antibodies. Data are presented as mean ± SEM. *p < 0.05, **p < 0.01, ***p < 0.001, Student's *t* test (A,C,D) and 1‐way ANOVA (B,G).

To examine metabolic function of adipose Mettl14, we placed *Mettl14^Δfat^* and *Mettl14^f/f^* male mice on HFD for 12 weeks and performed GTT and ITT. Overnight‐fasted plasma insulin levels were significantly lower in *Mettl14^Δfat^* than in *Mettl14^f/f^* mice (**Figure** **3**A). Blood glucose levels were significantly lower in *Mettl14^Δfat^* mice relative to *Mettl14^f/f^* mice after glucose (in GTT) and insulin (in ITT) injections (Figure 3B). To assess insulin signaling, male mice were fed HFD for 16 weeks, fasted overnight, and injected with insulin for 5 min (0.75 unit kg^−1^ body weight). Insulin stimulated phosphorylation of hepatic Akt (pThr308 and pSer473) to a significantly higher level in *Mettl14^Δfat^* than in *Mettl14^f/f^* mice (Figure 3C). Liver weight and liver triacylglycerol (TAG) content were significantly lower in *Mettl14^Δfat^* than in *Mettl14^f/f^* mice (Figure 3D,E). Hepatocyte lipid droplets, as assessed by either H&E or Oil red O staining of liver sections, were smaller and less abundant in *Mettl14^Δfat^* than in *Mettl14^f/f^* mice (Figure 3F). To examine influence of adipose development on the phenotypes, we treated adult *Mettl14^Δfat‐Tam^* and *Mettl14^f/f‐Tam^* (control) male mice (8 weeks old) with tamoxifen to specifically delete adipose *Mettl14* as described before. One week later, the mice were fed a HFD for 8 weeks and then subjected to GTT and ITT. Glucose intolerance and insulin resistance were markedly improved in *Mettl14^Δfat‐Tam^* mice compared to *Mettl14^f/f‐Tam^* mice (Figure 3G). Insulin stimulated phosphorylation of hepatic Akt (pThr308 and pSer473) to a higher level in *Mettl14^Δfat‐Tam^* than in *Mettl14^f/f‐Tam^* mice (Figure 3H). Liver steatosis was mitigated in *Mettl14^Δfat‐Tam^* mice (Figure S2C, Supporting Information). Taken together, these results suggest that aberrant upregulation of adipose Mettl14 and m6A modification increases risk to both obesity and obesity‐associated insulin resistance and NAFLD.

### 2.4 Adipocyte‐Specific Deletion of Mettl14 Blocks Brown Adipose Tissue (BAT) Thermogenesis

Brown adipocyte‐specific deletion of *Mettl3* impairs BAT thermogenesis,^[^ ^18^ ^]^ prompting us to examine BAT of *Mettl14^Δfat^* mice. Brown adipocyte lipid droplets were dramatically larger in *Mettl14^Δfat^* than in *Mettl14^f/f^* male mice on HFD for 16 weeks (Figure S3A, Supporting Information). BAT *Ucp1* mRNA and protein levels were dramatically lower in *Mettl14^Δfat^* mice (Figure S3B,C, Supporting Information). Upon cold exposure (4 °C), the body core temperature dropped to a larger degree in *Mettl14^Δfat^* than in *Mettl14^f/f^* littermates (Figure S3D, Supporting Information). Likewise, *Mettl14^Δfat^* female mice also displayed more severe whitening of BAT than *Mettl14^f/f^* females on HFD (Figure S3E, Supporting Information). BAT Ucp1 was downregulated in *Mettl14^Δfat^* females compared to *Mettl14^f/f^* females, but to a less degree relative to *Mettl14^Δfat^* males (Figure S3F, Supporting Information). Notably, BAT whitening was also observed in chow‐fed *Mettl14^Δfat^* male mice at 8 weeks of age (Figure S3G, Supporting Information). BAT dysfunctions in *Mettl14^Δfat^* mice prompted us to assess energy balance using CLAMS. To exclude obesity influence, we performed the assays on *Mettl14^f/f^* and *Mettl14^Δfat^* male mice before body weight divergence (Figure S4A, Supporting Information). Food intake was slightly lower in *Mettl14^Δfat^* mice (not statistically different) (Figure S4B, Supporting Information). O_2_ consumption and CO_2_ production (normalized to lean mass) were comparable between *Mettl14^f/f^* and *Mettl14^Δfat^* mice (Figure S4C,D, Supporting Information). Respiratory exchange ratio (RER) was lower in *Mettl14^Δfat^* mice (Figure S4C,D, Supporting Information), suggesting that *Mettl14^Δfat^* animals use lipids as metabolic fuel. Indeed, whole body fatty acid β oxidation, but not glucose oxidation, was higher in *Mettl14^Δfat^* than in *Mettl14^f/f^* mice (Figure S4E, Supporting Information). Body core temperature was comparable between *Mettl14^f/f^* and *Mettl14^Δfat^* mice (Figure S4F, Supporting Information). Feces weights, fecal TAG levels, and absorption of orally administrated olive oil were comparable between *Mettl14^f/f^* and *Mettl14^Δfat^* mice (Figure S4G,H, Supporting Information). Thus, obesity protection cannot be explained by BAT thermogenesis and food intake and absorption in *Mettl14^Δfat^* mice.

### 2.5 Adipose Mettl14 Cell‐Autonomously Suppresses Adipocyte Lipolysis

**Figure 4:**
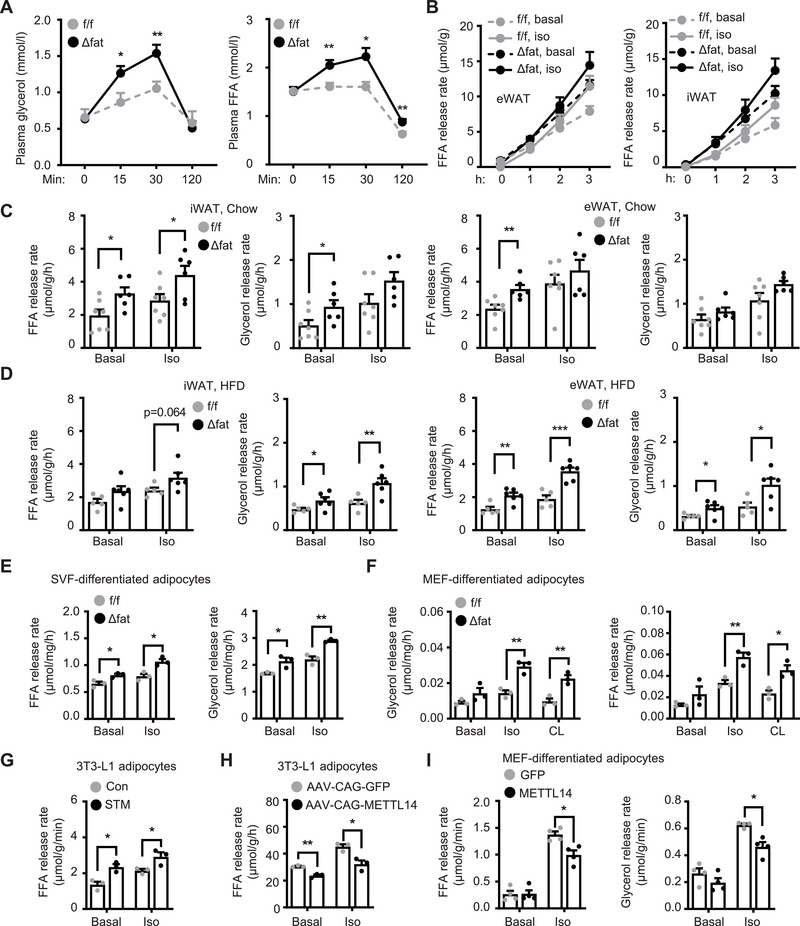
Mettl14 cell‐autonomously inhibits lipolysis in adipocytes. A) Male mice (8 weeks) were fed a high fat diet (HFD) for 4 weeks, fasted for 4 h, and injected with isoproterenol (Iso, 10 mg kg^−1^ body weight). Plasma glycerol and FFA levels were measured. Mettl14f/f: *n* = 7, Mettl14Δfat: *n* = 9. B,C) Inguinal white adipose tissue (iWAT) and epididymal WAT (eWAT) were harvested from chow‐fed mice (6–8 weeks old) and stimulated with isoproterenol (1 × 10^−6^ m) for 3 h. Glycerol and FFA secretion rates were measured and normalized to WAT weights. iWAT Mettl14f/f: *n* = 7, iWAT Mettl14Δfat: *n* = 6, eWAT Mettl14f/f: *n* = 7, eWAT Mettl14Δfat: *n* = 6. D) Male mice (8 weeks) were fed a HFD for 4 weeks. iWAT and eWAT were harvested to measure lipolysis rates under basal and isoproterenol‐stimulated conditions. Mettl14f/f: *n* = 5, Mettl14Δfat: *n* = 6. E) Stromal vascular fraction (SVF) cells were isolated from eWAT, differentiated into adipocytes, and stimulated with isoproterenol (1 × 10^−6^ m) for 3 h. Glycerol and FFA secretion rates were measured and normalized to protein levels (*n* = 3 repeats per group). F) Mouse embryonic fibroblasts (MEFs) were differentiated into adipocytes and stimulated with isoproterenol (1 × 10^−6^ m) or CL316243 (1 × 10^−6^ m, CL) for 3 h. Glycerol‐ and FFA‐releasing rates were measured and normalized to protein levels (*n* = 3 repeats per group). G) 3T3‐L1 cell were differentiated into adipocytes, pretreated with STM2457 (5 × 10^−6^ m) for 48 h, and then stimulated with isoproterenol (1 × 10^−6^ m) for 60 min. FFA‐releasing rates were measured (*n* = 3 repeats per group). H) 3T3‐L1 adipocytes were transduced with AAV‐CAG‐METTL14 or AAV‐CAG‐GFP vectors and stimulated with isoproterenol (1 × 10^−6^ m) for 60 min. FFA‐releasing rates were measured and normalized to protein levels (*n* = 3 repeats per group). I) MEFs were transduced with METTL14 or GFP lentiviral vectors, differentiated into adipocytes, and stimulated with isoproterenol (1 × 10^−6^ m) for 3 h. Glycerol‐ and FFA‐releasing rates were measured and normalized to protein levels (*n* = 4 repeats per group). Data are presented as mean ± SEM. *p < 0.05, **p < 0.01, ***p < 0.001, Student's *t* test (C‐I) and 1‐way ANOVA (A).

Increased fatty acid β oxidation prompted us to examine adipose lipolysis and lipid mobilization in *Mettl14^Δfat^* mice. We placed *Mettl14^Δfat^* and *Mettl14^f/f^* mice on HFD for 4 weeks (increasing adipose Mettl14 expression) and then injected them with β adrenergic agonist isoproterenol. Blood glycerol and FFA levels were significantly higher in *Mettl14^Δfat^* than in *Mettl14^f/f^* mice within 30 min after injection (**Figure** **4**A). To directly measure lipolysis ex vivo, we dissected iWAT and eWAT from *Mettl14^Δfat^* and *Mettl14^f/f^* mice (8 weeks on chow diet) and stimulated them with isoproterenol (1 × 10^−6^ m) for 3 h. Glycerol‐ and FFA‐releasing rates were significantly higher in *Mettl14^Δfat^* than in *Mettl14^f/f^* iWAT and eWAT under both baseline and isoproterenol‐stimulated conditions (Figure 4B,C). Given that lipolysis is blunted in obesity, we placed *Mettl14^Δfat^* and *Mettl14^f/f^* male littermates on HFD for 4 weeks. Likewise, glycerol‐ and FFA‐releasing rates of iWAT and eWAT were also substantially higher in *Mettl14^Δfat^* mice relative to *Mettl14^f/f^* mice (Figure 4D). Considering that insulin increases m6A methylation, we tested if Mettl14 deficiency impairs insulin suppression of lipolysis. We isolated eWAT and iWAT from chow‐fed mice and stimulated them with isoproterenol in the presence or absence of insulin. Insulin decreased lipolysis rates in both eWAT and iWAT from *Mettl14^f/f^*, but not *Mettl14^Δfat^*, mice (Figure S5A, Supporting Information). As expected, isoproterenol stimulated lipolysis to a higher level in *Mettl14^Δfat^* than in *Mettl14^f/f^* mice. To corroborate these results, we isolated SVF cells from WAT, differentiated them into adipocytes in vitro, stimulated adipocytes with isoproterenol (1 × 10^−6^ m) for 3 h, and measured glycerol and FFA releases into culture medium. Adipocyte differentiation was comparable between *Mettl14^Δfat^* and *Mettl14^f/f^* SVF cells. Glycerol‐ and FFA‐releasing rates were significantly higher in *Mettl14^Δfat^* than in *Mettl14^f/f^* adipocytes under both baseline and isoproterenol‐stimulated conditions (Figure 4E). To further confirm these findings, we prepared mouse embryonic fibroblasts (MEFs) from *Mettl14^Δfat^* and *Mettl14^f/f^* embryos, differentiated them into adipocytes in vitro, and stimulated adipocytes with isoproterenol or CL316243 (a β3 adrenergic receptor agonist) for 3 h. Adipocyte differentiation and TAG levels were comparable between *Mettl14^Δfat^* and *Mettl14^f/f^* MEFs (Figure S5B, Supporting Information). Glycerol‐ and FFA‐releasing rates were significantly higher in *Mettl14^Δfat^* than in *Mettl14^f/f^* adipocytes under baseline, isoproterenol‐stimulated, and CL316243‐stimulated conditions (Figure 4F). Given that Mettl14 binds to Mettl3 to assemble the functional m6A methyltransferase complex, we reasoned that pharmacological inhibition of Mettl3 might enhance lipolysis. 3T3‐L1 preadipocytes were differentiated into adipocytes, pretreated for 48 h with METTL3 inhibitor STM2457,^[^ ^19^ ^]^ and then stimulated with isoproterenol for 60 min. STM2457 pretreatment increased FFA‐releasing rates under both baseline and isoproterenol‐stimulated conditions (Figure 4G). To test if METTL14 overexpression has the opposite effect, we transduced 3T3‐L1 adipocyte cultures with AAV‐CAG‐METTL14 (human) or AAV‐CAG‐green fluorescent protein (GFP) vectors, stimulated them with isoproterenol (1 × 10^−6^ m), and then assessed lipolysis. Human METTL14 and mouse Mettl14 share over 99% of identical amino acids (Figure S5C, Supporting Information). We confirmed that METTL14 was overexpressed in AAV‐CAG‐METTL14‐transduced cells (Figure S5D, Supporting Information). METTL14 overexpression decreased FFA‐releasing rates under both baseline and isoproterenol‐stimulated conditions (Figure 4H). To further confirm these results, we transduced primary MEFs with METTL14 or GFP lentiviral vectors and differentiated them into adipocytes. METTL14 was modestly overexpressed in METTL14‐transduced cells (Figure S5E, Supporting Information). METTL14 overexpression decreased glycerol‐ and FFA‐releasing rates under both baseline and isoproterenol‐stimulated conditions (Figure 4I). These results unveil adipose Mettl14 as a previously unrecognized lipolysis suppressor. It is likely that increased lipolysis may explain, at least in part, obesity protection in *Mettl14^Δfat^* mice.

### 2.6 Mettl14 Downregulates Adrb2/3 and Lipolysis Machinery Components in Adipocytes

**Figure 5:**
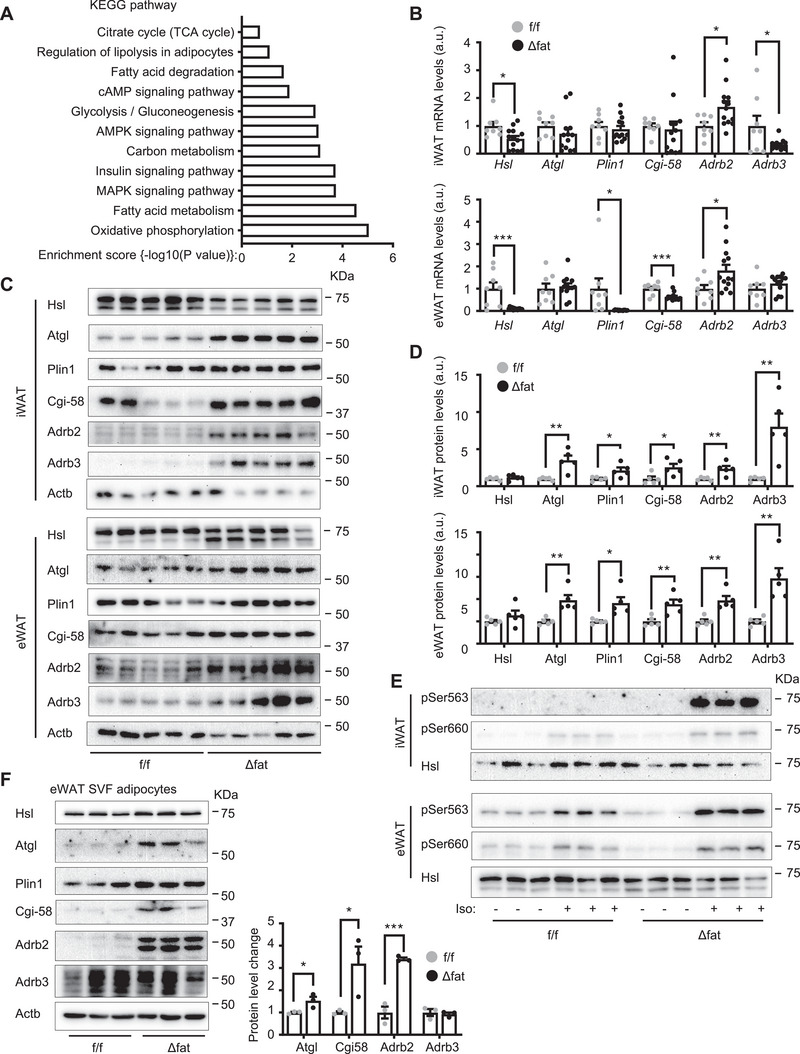
Mettl14 downregulates Adrb2, Adrb3, Atgl, and Cgi‐58 and inhibits β adrenergic signaling in adipocytes. A–D) Mettl14f/f and Mettl14Δfat males (8 weeks) were fed a high fat diet (HFD) for 16 weeks, and inguinal white adipose tissue (iWAT) and epididymal WAT (eWAT) were isolated. A) eWAT was used for RNA‐seq. Differentially expressed genes were analyzed by KEGG (*n* = 3 mice per group). B) mRNA abundance was measured by qPCR and normalized to 36B4 levels. a.u.: arbitrary unit. Mettl14f/f: *n* = 8, Mettl14Δfat: *n* = 13. C,D) iWAT and eWAT extracts were immunoblotted with the indicated antibodies. Protein levels were quantified and normalized to Actb levels (*n* = 5 mice per group). E) Male mice (8 weeks) were fed a HFD for 4 weeks. iWAT and eWAT were isolated and stimulated with isoproterenol (1 × 10^−6^ m) for 15 min. WAT extracts were immunoblotted with the indicated antibodies. F) SVF cells were isolated from eWAT and differentiated into adipocytes. Cell extracts were immunoblotted with the indicated antibodies. Protein levels were normalized to Actb levels (*n* = 3 repeats per group). Data are presented as mean ± SEM. *p < 0.05, **p < 0.01, ***p < 0.001, Student's *t* test.

We next aimed to identify target transcripts of Mettl14 involved in lipolysis. To increase adipose Mettl14 expression, we placed *Mettl14^Δfat^* and *Mettl14^f/f^* male mice on a HFD for 20 weeks and then isolated eWAT for RNA‐seq analysis. We identified 4481 genes upregulated and 3486 genes downregulated in *Mettl14^Δfat^* mice (by >1.2 folds, *p* < 0.08) (GSE233782). Gene ontology (GO) analysis of the differentially expressed genes revealed that Mettl14 deficiency affected multiple pathways, including citrate cycle, lipolysis, fatty acid degradation, and cAMP signaling pathways (**Figure** **5**A). Fatty acid metabolic processes and lipid oxidation were augmented in *Mettl14^Δfat^* eWAT (Figure S6A, Supporting Information). Heatmap analysis demonstrated that adipose expressions of genes involved in β adrenergic signaling, lipolysis, and fatty acid β oxidation were higher in *Mettl14^Δfat^* than in *Mettl14^f/f^* mice (Figure S6B, Supporting Information). qPCR analysis confirmed upregulation of Adrb2, Cpt1b, and PPARα in *Mettl14^Δfat^* mice (Figure 5B and Figure S6C, Supporting Information). Expressions of cytokines Il6 and Tnfα were lower in *Mettl14^Δfat^* mice (Figure S6C, Supporting Information), suggesting that adipose immune microenvironment is improved. In contrast to BAT where Ucp1 is suppressed, in iWAT and eWAT, Ucp1 expression was significantly higher in *Mettl14^Δfat^* than in *Mettl14^f/f^* mice (Figure S6C, Supporting Information). Therefore, Mettl14 deficiency exerts the opposing actions in BAT and beige fat. It is likely that increased beige fat activity in eWAT and iWAT oxidizes lipolysis‐generated free fatty acids, contributing to protection of *Mettl14^Δfat^* mice against obesity.

To examine the impact of Mettl14 deficiency on β adrenergic signaling and the lipolysis machinery, we placed *Mettl14^f/f^* and *Mettl14^Δfat^* mice on a HFD for 16 weeks (increasing adipose Mettl14 expression in *Mettl14^f/f^* mice) and measured Adrb2, Adrb3, Atgl, and Cgi‐58 levels in iWAT and eWAT extracts by immunoblotting. Adrb2, Adrb3, Atgl, and Cgi‐58 levels in both iWAT and eWAT were significantly higher in *Mettl14^Δfat^* than in *Mettl14^f/f^* mice (Figure 5C,D). *Adrb3*, *Atgl*, and *Cgi‐58* mRNA levels were not increased in *Mettl14^Δfat^* mice (Figure 5B), suggesting that Adrb3, Atgl, and Cgi‐58 are upregulated by a posttranscriptional mechanism. Next, we stimulated iWAT and eWAT explants with isoproterenol (1 × 10^−6^ m) for 15 min and immunoblotted WAT extracts with anti‐phospho‐Hsl antibodies (pSer563, pSer660). Isoproterenol stimulated Hsl phosphorylation (pSer563, pSer660) to a substantially higher level in *Mettl14^Δfat^* than in *Mettl14^f/f^* mice (Figure 5E and Figure S7A, Supporting Information). Isoproterenol also increased phosphorylation of PKA pan‐substrates in iWAT and eWAT to a markedly higher level in *Mettl14^Δfat^* than in *Mettl14^f/f^* mice (Figure S7B, Supporting Information). To further test if Mettl14 cell‐autonomously regulates β adrenergic receptors and lipolysis machinery components, we isolated SVF cells from eWAT (or MEFs) and differentiated them into adipocytes in vitro. Adrb2, Atgl, and Cgi‐58 protein levels were substantially higher in *Mettl14^Δfat^* adipocytes while *Adrb2*, *Atgl*, and *Cgi‐58* mRNA levels were comparable between *Mettl14^Δfat^* and *Mettl14^f/f^* adipocytes (Figure 5F and Figure S7C, Supporting Information). This further supports the notion that Mettl14 deficiency upregulates these proteins by a posttranscriptional mechanism. To verify these findings, MEFs were differentiated into adipocytes in vitro and stimulated with isoproterenol. Isoproterenol stimulated phosphorylation of Hsl (pSer563, pSer660) and PKA pan‐substrates to a markedly higher level in *Mettl14^Δfat^* than in *Mettl14^f/f^* adipocytes (Figure S7D, Supporting Information). To test if RNA m6A methylation is involved, we treated 3T3‐L1 adipocytes with Mettl3 inhibitor STM2457 for 48 h. STM2457 considerably increased Atgl and Cgi‐58 protein levels (Figure S7E, Supporting Information). Collectively, these results suggest that aberrant upregulation of adipose Mettl14 and m6A modification contributes to adipose catecholamine resistance and lipolysis suppression in obesity.

### 2.7 Mettl14 Inhibits Adrb2, Adrb3, Atgl, and Cgi‐58 Translations via Promoting m6A Methylation

**Figure 6:**
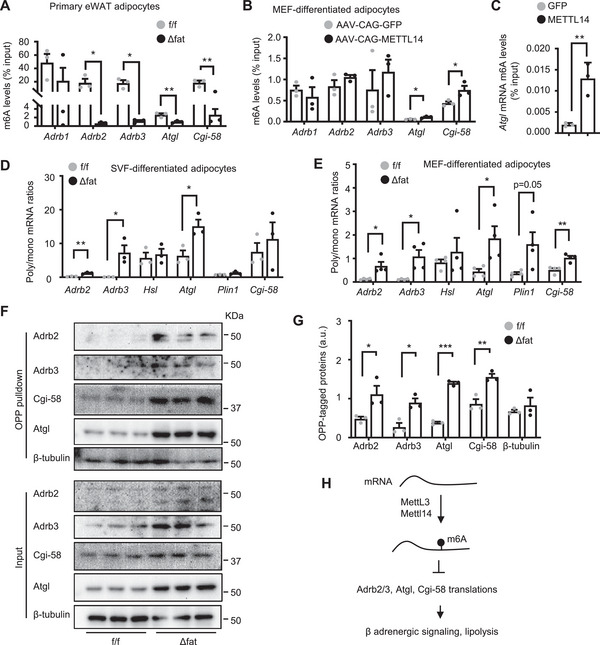
Ablation of Mettl14‐elicited m6A methylation enhances translation of Adrb2, Adrb3, Atgl, and Cgi‐58 transcripts in adipocytes. A) Primary adipocytes were isolated from epididymal white adipose tissue (eWAT) (8 weeks old). m6A content in individual transcripts was measured by m6A RNA immunoprecipitation (RIP) and normalized to transcript inputs (*n* = 3 mice per group). B) Mouse embryonic fibroblasts (MEFs) were differentiated into adipocytes and transduced with AAV‐CAG‐METLL14 or AAV‐CAG‐METTL14 vectors. m6A content in individual transcripts was measured by m6A RIP (*n* = 3 repeats per group). C) Atgl plasmids were cotransfected with METTL14 plasmids into HEK293 cells. m6A content of Atgl transcripts was measured by m6A RIP (*n* = 3 repeats per group). D) Stromal vascular fraction (SVF) cells were isolated from Inguinal WAT (iWAT), differentiated into adipocytes, and subjected to polysome analysis. Polysome (poly) to monosome (mono) associated mRNA was quantified by qPCR to calculate poly/mono ratio (*n* = 3 repeats per group). E) MEFs were differentiated into adipocytes and subjected to polysome analysis. Poly/mono mRNA ratio was presented (*n* = 4 repeats per group). F,G) MEFs were differentiated into adipocytes and subjected to *O*‐propargyl‐puromycin (OPP) pulldown assays. F) Nascent OPP‐tagged proteins were pulled down and immunoblotted with the indicated antibodies. G) OPP‐tagged Atgl, Cgi‐58, Adrb2, and Adrb3 were quantified and normalized to input Atgl, Cgi‐58, Adrb2, and Adrb3 levels, respectively (*n* = 3 repeats). H) Mettl3/Mettl14 complex install m6A in Adrb2, Adrb3, Atgl, and Cgi58 transcripts and inhibit their translation. These suppress β adrenergic signaling and lipolysis. Data are presented as mean ± SEM. *p < 0.05, **p < 0.01, ***p < 0.001, Student's *t* test.

We postulated that Mettl14 might assemble the Mettl14/Mettl3 RNA transferase complex (m6A writer) to install m6A in *Adrb2*, *Adrb3*, *Atgl*, and *Cgi‐58* transcripts. We isolated primary adipocytes from eWAT and measured m6A content using RNA immunoprecipitation (RIP) assays and anti‐m6A antibody (IgG as control). The m6A contents of *Adrb2*, *Adrb3, Atgl*, and *Cgi‐58*, but not *Adrb1*, transcripts were substantially lower in *Mettl14^Δfat^* than in *Mettl14^f/f^* mice (**Figure** **6**A). IgG was unable to precipitate the transcripts (Figure S8A, Supporting Information). To test if overexpression of Mettl14 has the opposite effects, MEFs were prepared from *Mettl14^f/f^* mice, differentiated into adipocytes, and transduced with AAV‐CAG‐METTL14 or AAV‐CAG‐GFP. We confirmed METTL14 overexpression in AAV‐CAG‐METTL14‐transduced cells (Figure S8B, Supporting Information). Overexpression of METTL14 increased *Atgl* and *Cgi‐58* transcript m6A contents (Figure 6B). The m6A levels of *Adrb2* and *Adrb3* transcripts were not statistically different between the two groups, likely due to modest overexpression of METTL14. To further confirm that Mettl14 directly mediates m6A methylation, we cotransfected METTL14 plasmids with Atgl plasmids into HEK293 cells. METTL14 dramatically increased *Atgl* mRNA m6A content (Figure 6C).

Given that m6A affects mRNA decay,^[^ ^20^ ^]^ we tested if Mettl14 regulates *Atgl*, *Cgi‐58*, *Adrb2*, and *Adrb3* transcript stability using transcription inhibitor actinomycin D. We isolated SVF cells from iWAT, differentiated them into adipocytes, treated adipocytes with actinomycin D, and measured mRNA abundance by qPCR. *Atgl*, *Adrb2*, and *Adrb3* mRNA decays were comparable between *Mettl14^Δfat^* and *Mettl14^f/f^* adipocytes, whereas *Cgi‐58* mRNA decay was slightly higher in *Mettl14^Δfat^* adipocytes (Figure S8C, Supporting Information).

To test if Mettl14‐mediated m6A suppresses *Atgl*, *Cgi‐58*, *Adrb2*, and *Adrb3* translations, we performed polysome profiling assays in SVF (from iWAT) differentiated adipocytes. Polysome (a mark of increased translation) to monosome ratios of *Adrb2*, *Adrb3*, *Atgl*, and *Cgi‐58* transcripts were significantly higher in *Mettl14^Δfat^* than in *Mettl14^f/f^* adipocytes (Figure 6D). Likewise, in MEF‐differentiated adipocytes, polysome to monosome ratios of *Adrb2*, *Adrb3*, *Atgl*, and *Cgi‐58* were also significantly higher in *Mettl14^Δfat^* than in *Mettl14^f/f^* cells (Figure 6E). To complement these studies, we measured protein synthesis using *O*‐propargyl‐puromycin (OPP) pulldown assays.^[^ ^21^ ^]^ We treated MEF‐differentiated adipocytes with OPP to tag newly translated proteins with OPP (Figure S8D, Supporting Information). OPP‐tagged proteins were purified by biotin/streptavidin pulldown and immunoblotted with antibodies to Atgl, Cgi‐58, Adrb2, and Adrb3. Translational rates of Adrb2, Adrb3, Atgl, and Cgi‐58, but not β‐tubulin, were markedly higher in *Mettl14^Δfat^* than in *Mettl14^f/f^* adipocytes (Figure 6F,G). These results suggest that Mettl14‐induced m6A methylation inhibits the translational capability of *Atgl*, *Cgi‐58*, *Adrb2*, and *Adrb3* transcripts, thereby suppressing β adrenergic signaling and lipolysis in WAT (Figure 6H).

### 2.8 Aberrant Mettl14/m6A/Translation Axis Contributes to Adipose Catecholamine Resistance and Lipolysis Suppression in Obesity

**Figure 7:**
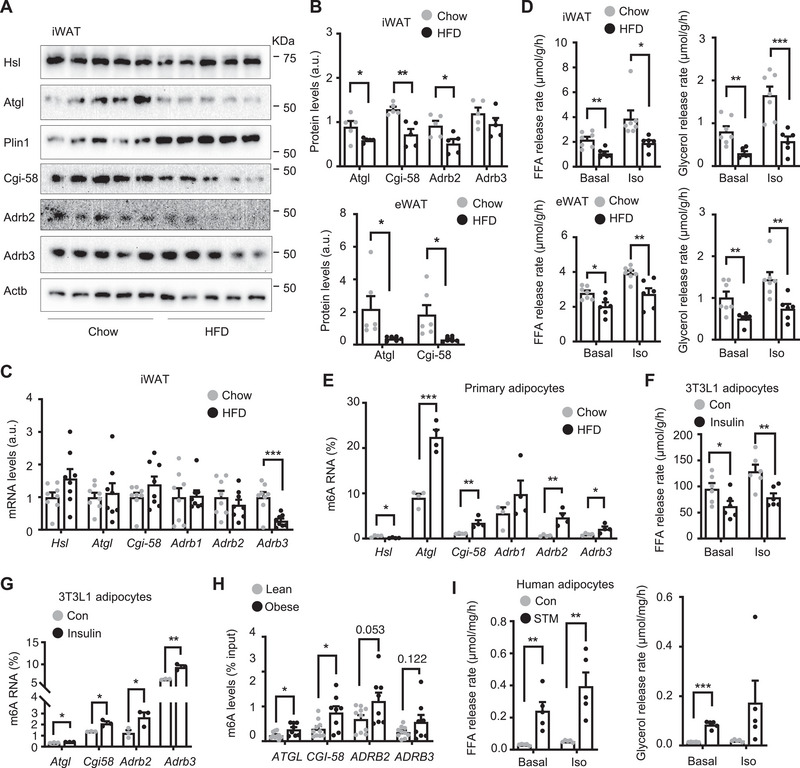
Mettl14/m6A‐based epitranscriptomic reprogramming contributes to adipose catecholamine resistance and lipolysis suppression in obesity. A–E) C57BL/6J male mice (8 weeks) were on high fat diet (HFD) for 12 weeks. A,B) Inguinal white adipose tissue (iWAT; *n* = 5 per group) and epididymal WAT (eWAT) (*n* = 6 per group) extracts were immunoblotted with the indicated antibodies. Protein levels were quantified and normalized to Actb levels. C) iWAT mRNA was measured by qPCR and normalized to 36B4 levels (*n* = 8 mice per group). D) iWAT and eWAT explants were stimulated with 1 × 10^−6^ m isoproterenol (Iso) for 3 h, and lipolysis was measured and normalized to WAT weight. Chow: *n* = 7, HFD: *n* = 6. E) Primary adipocytes were purified from eWAT. m6A content in individual transcripts was measured in adipocytes using m6A RNA immunoprecipitation (RIP) assays and normalized to input (*n* = 4 mice per group). F,G) 3T3‐L1 cells were differentiated into adipocytes. F) 3T3‐L1 adipocytes were stimulated with 1 × 10^−6^ m isoproterenol and FFA‐releasing rates were measured and normalized to protein levels (*n* = 6 repeats per group). G) m6A content in individual transcripts were measured by m6A RIP and normalized to input (*n* = 3 repeats per group). H) m6A content was measured in human visceral WAT using m6A RIP assays (normalized to input). Lean: *n* = 10, obese: *n* = 8. I) Stromal vascular fraction (SVF) cells were isolated from human visceral fat, differentiated into adipocytes, pretreated with STM2457 (2.5 × 10^−6^ m) for 48 h, and stimulated with isoproterenol (1 × 10^−6^ m) for 120 min. Glycerol‐ and FFA‐releasing rates were measured and normalized to protein levels (*n* = 5 subjects per group). Data are presented as mean ± SEM. *p < 0.05, **p < 0.01, ***p < 0.001, Student's *t* test.

Adipose Mettl3, Mettl14, and m6A levels are upregulated in obesity, prompting us to test if their molecular targets involved in lipolysis are downregulated in WAT. We placed C57BL/6J male mice on a HFD for 12 weeks and harvested iWAT and eWAT for immunoblotting. Atgl in both iWAT and eWAT was significantly lower in HFD‐fed than in chow‐fed mice (**Figure** **7**A and Figure S9A, Supporting Information), confirming previous reports.^[^ ^22^ ^]^ Likewise, adipose Atgl, Cgi‐58, and Adrb2 protein levels were also lower in HFD than in chow fed mice (Figure 7A,B). *Adrb2*, *Atgl*, and *Cgi‐58* mRNA levels in iWAT were comparable between chow and HFD fed mice (Figure 7C), raising the possibility that Adrb2, Atgl, and Cgi‐58 proteins are downregulated in obesity by a posttranscriptional mechanism (e.g., m6A methylation). Adipose *Adrb3* mRNA levels were lower in HFD‐fed mice (Figure 7C and Figure S9B, Supporting Information). To directly assess lipolysis, we stimulated iWAT and eWAT explants with isoproterenol (1 × 10^−6^ m) for 3 h and measured lipolysis rate. Glycerol‐ and FFA‐releasing rates under both baseline and isoproterenol‐stimulated conditions were significantly lower in HFD than in chow fed mice (Figure 7D and Figure S9C, Supporting Information). To test the role of m6A modification, we purified primary adipocytes from eWAT and measured m6A contents of *Atgl*, *Cgi‐58*, *Adrb2*, and *Adrb3* transcripts using m6A RIP‐qPCR. Adipocyte *Atgl*, *Cgi‐58*, *Adrb2*, and *Adrb3* m6A contents were significantly higher in HFD‐fed than in chow‐fed mice (Figure 7E). Considering that obesity is associated with hyperinsulinemia, we tested if insulin stimulates m6A methylation in these transcripts. As expected, insulin stimulation suppressed lipolysis in 3T3‐L1 adipocytes (Figure 7F). Insulin also markedly increased m6A contents of *Atgl*, *Cgi‐58*, *Adrb2*, and *Adrb3* transcripts (Figure 7G). Insulin did not affect total mRNA levels of these transcripts under these conditions (Figure S9D, Supporting Information). To test if adipose METTL14 similarly regulates lipolysis in human obesity, we measured expression of METTL14 expression and m6A levels in visceral WAT. We did not detect significant difference of *METTL14* mRNA levels between the lean and the obese groups due to a large variation in the obese samples (Figure S9E, Supporting Information). METTL14 proteins were higher in the obese than in the lean WAT samples (Figure S9F, Supporting Information). Importantly, m6A contents of *ATGL* and *CGI‐58* transcripts were significantly higher in the obese than in the lean WAT samples (Figure 7H). *ADRB2/3* transcript m6A contents were also higher in the obese group (not statistically different). It is worth mentioning that some WAT samples were stored for over 7 years during which m6A may be erased. To further test m6A in human adipocytes, we isolated SVF cells from human visceral WAT biopsies, differentiated them into adipocytes, treated human adipocytes with METTL3 inhibitor STM2457, and then measured lipolysis in the presence or absence of β adrenergic stimulation. STM2457 inhibition of m6A modification markedly increased FFA‐ and glycerol‐releasing rates under both baseline and isoproterenol‐stimulated conditions (Figure 7I). Collectively, these results suggest that upregulation of adipocyte Mettl3, Mettl14, and m6A methylation of *Atgl*, *Cgi‐58*, *Adrb2*, and *Adrb3* transcripts causes, at least in part, adipose catecholamine resistance, lipolysis suppression, adipose expansion, and metabolic disorders in obesity.

## 3 Discussion

Mettl14 heterodimerizes with Mettl3 to assemble the m6A writer that installs m6A methylation on target RNA transcripts. Here, we delineate a previously unrecognized Mettl14‐based epitranscriptomic remodeling governing β adrenergic signaling, lipolysis, and WAT growth. Deletion of *Mettl14* decreased, whereas overexpression of METTL14 increased, the m6A contents of *Adrb2*, *Adrb3*, *Atgl*, and *Cgi‐58* transcripts in adipocytes. These suggest that the Mettl14/Mettl3 writer directly deposits the m6A code on transcripts controlling β adrenergic signaling (*Adrb2* and *Adrb3*) and lipolysis (*Atgl* and *Cgi‐58*). Mettl14‐mediated m6A methylation decreased translation of these transcripts, as measured by polysome profiling and OPP assays, thereby inhibiting β adrenergic signaling and lipolysis in WAT. Like genetic ablation of Mettl14, Mettl3 inhibitor‐induced blocking of m6A depositions also increased lipolysis in 3T3‐L1 adipocytes and human adipocytes. These findings further support the concept that Mettl14‐elicited m6A methylation of *Adrb2*, *Adrb3*, *Atgl*, *Cgi‐58*, and perhaps additional transcripts act as a molecular brake to restrain β adrenergic signaling, lipolysis, and lipid mobilization and oxidation. Collectively, these findings define a m6A‐centric RNA modification paradigm shaping adipose signaling, lipolysis, and growth.

We found that adipose Mettl3, Mettl4, and m6A levels were upregulated in obesity. Consistently, the m6A levels of adipose *Adrb2*, *Adrb3*, *Atgl*, and *Cgi‐58* transcripts were substantially increased in obesity in both mice and humans. Further supporting the concept that m6A modification inhibits translation, adipose Adrb2, Adrb3, Atgl, and Cgi‐58 protein levels were decreased in obese mice. It has been long recognized that obesity is associated with adipose catecholamine resistance and lipolysis suppression in both mice and humans in WAT.^[^ ^1^, ^8^ ^]^ We confirmed that adipose lipolysis, under both baseline and β adrenergic‐stimulated conditions, was substantially lower in mice with HFD‐induced obesity. However, the underlying molecular mechanism for the obesity‐linked adipose defects remains poorly understood. The findings of this work raise the intriguing possibility that aberrant upregulation of adipose Mettl3, Mettl14, and m6A methylation of *Adrb2*, *Adrb3*, *Atgl*, and *Cgi‐58* transcripts, and translational suppression of these transcripts may explain adipose catecholamine resistance and lipolysis suppression in obesity. Obesogenic factors responsible for upregulation of Mettl3 and Mettl14 are currently unknown. Of note, insulin stimulation increased Mettl4 and m6A content in adipocytes. Insulin is known to suppress lipolysis by counteracting β adrenergic signaling and suppressing Atgl transcription.^[^ ^1^, ^23^ ^]^ The Mettl14/m6A pathway provides an additional mechanism for insulin to inhibit β adrenergic signaling and lipolysis. Notably, obesity is associated with hyperinsulinemia and perhaps, elevated insulin levels are involved in upregulation of adipose Mettl14 and m6A modifications in obesity. However, obesity is also linked to insulin resistance, impeding insulin response. Additional studies are needed to assess the contributions of hyperinsulinemia and other obesogenic factors to upregulation of adipose Mettl3, Mettl14, and m6A‐based epitranscriptomic remodeling in obesity. Importantly, adipocyte‐specific deletion of *Mettl14* blocked HFD‐induced obesity, insulin resistance, glucose intolerance, and NAFLD, accompanied by increased β adrenergic signaling and lipolysis in WAT. The phenotypes of *Mettl14^Δfat^* mice provide proof‐of‐concept evidence that Mettl14‐based epitranscriptomic inhibition of adipose β adrenergic signaling and lipolysis is a causal factor for obesity and its associated metabolic disorders. Lipolysis suppression is expected to increase excessive lipid storage and WAT expansion. In line with this notion, transgenic expression of Atgl in adipocytes alleviates obesity and metabolic disorders.^[^ ^2a^ ^]^ Therefore, the adipose Mettl14/m6A pathway may serve as a potential therapeutic target for treatment of obesity and related metabolic disease.

We observed that *Mettl14^Δfat^* mice displayed severe whitening and thermogenic dysfunction of BAT. Given that *Mettl14* is also deleted in brown adipocytes of *Mettl14^Δfat^* mice, we postulate that BAT‐intrinsic deficiency of Mettl14 cell‐autonomously impairs BAT functions. In line with this idea, BAT‐specific deletion of *Mettl3*, using *Ucp1‐Cre* drivers, similarly induces BAT whitening and dysfunctions.^[^ ^18^ ^]^ BAT paucity is known to increase beige fat growth in compensation.^[^ ^24^ ^]^ Indeed, beige fat activity, as assessed by expression of adipocyte‐selective genes (*Ucp1*, *Pparα*, and *Pgc1α*) in WAT, was higher in *Mettl14^Δfat^* relative to *Mettl14^f/f^* littermates. Recently, lipolysis‐derived linoleic acid was reported to stimulate beige progenitor cell proliferation and beige fat recruitment in mice.^[^ ^25^ ^]^ Given that adipose lipolysis is elevated in *Mettl14^Δfat^* mice, beige fat‐activating lipolytic products are expected to be increased, thus further supporting beige fat recruitment. The opposite phenotypes of BAT and beige fat in *Mettl14^Δfat^* mice suggest that Mettl14 exerts the opposing actions on these two cell types determined by their distinct intracellular microenvironments. Unlike *Mettl14^Δfat^* mice which are resistant to HFD‐induced obesity, mice with BAT‐specific deletion of *Mettl3* are prone to diet‐induced obesity.^[^ ^18^ ^]^ These opposite phenotypes further support the notion that increases in β adrenergic signaling and lipolysis in WAT, which are observed in *Mettl14^Δfat^* mice but not in Mettl3‐deficient mice, mediate protection of *Mettl14^Δfat^* mice against obesity. In *Mettl14^Δfat^* mice, global fatty acid β oxidation was increased to consume free fatty acids derived from adipose lipolysis, thereby counteracting lipid trafficking into the liver and liver steatosis in *Mettl14^Δfat^* mice. In conclusion, we propose a Mettl14/m6A‐centric epitranscriptomic reprogramming paradigm in obesity (Figure S9G, Supporting Information). Obesogenic factors aberrantly upregulate adipose Mettl3 and Mettl14, which induce m6A methylation of *Adrb2*, *Adrb3*, *Atgl*, *Cgi‐58*, and perhaps additional transcripts to suppress their translations. These epitranscriptomic‐orchestrated RNA modifications in turn drive adipose catecholamine resistance and lipolysis suppression, leading to WAT expansion, obesity progression, and metabolic disease.

There are limitations in this study. Aside from *Adrb2*, *Adrb3*, *Atgl*, and *Cgi‐58* mRNAs, adipose Mettl14 and Mettl3 may target additional transcripts involved in adipose growth, and these putative targets are unknown. Additional evidence is needed to further confirm that m6A methylation mediates the Mettl14 action; m6A readers, which mediate the Mettl14 action, are needed to be identified. Aside from beige adipocytes, additional cell types consuming lipolysis‐derived fatty acids to protect against liver steatosis in *Mettl14^Δfat^* mice are needed to be identified.

## 4 Experimental Section

### Animals

Mice were housed on a 12 h light‐dark cycle at 25 °C and fed ad libitum a chow diet (9% fat; TestDiet, St. Louis, MO) or HFD (60% fat; Research Diets, New Brunswick, NJ). *Mettl14^f/f^* and *adiponectin‐Cre* mice were characterized previously.^[^ ^1^, ^15^, ^17^ ^]^ *Mettl14^f/f^* mice were crossed with *adiponectin‐Cre* (JAX stock number: 02 8020) or *adiponectin‐CreERT* (JAX stock number: 02 4671) drivers to obtain *Mettl14^Δfat^* (*Mettl14^f/f^;Cre^+/−^*) or *Mettl14^Δfat‐Tam^* (*Mettl14^Δfat‐Tam^;CreERT^+/−^*) mice. *Mettl14^Δfat‐Tam^* or *Mettl14^f/f^* (control) mice (8 weeks old) were intraperitoneally injected with tamoxifen (50 mg kg^−1^ body weight) three times at a 2 day‐interval to delete adipose *Mettl14*.

### Ethics Statements

Animal research complied with all relevant ethnical regulations. Animal experiments were conducted following the protocols approved by the University of Michigan Institutional Animal Care and Use Committee (IACUC).

### Plasma Insulin, GTT, ITT, Fat Content, and In Vivo Lipolysis

Blood samples were collected from tail veins. Plasma insulin was measured using insulin ELISA kits (CRYSTAL CHEM, Downers Grove, IL). For GTT, mice were fasted overnight and intraperitoneally injected with glucose (1.5 g kg^−1^). For ITT, mice were fasted for 6 h and intraperitoneally injected with insulin (0.75 units kg^−1^). Blood glucose was measured after injection. Fat content and lean body mass were measured using a dual‐energy X‐ray absorptiometry pDexa (Norland Stratec). Male mice (8 weeks old) were fed a HFD for 4 weeks, fasted for 4 h, and injected with isoproterenol (10 mg kg^−1^). Blood samples were collected via tail veins to measure plasma glycerol (Sigma, Free Glycerol Reagent F‐6428) and FFA (NEFA‐HR reagents, Wako Chemicals, Richmond, VA) concentrations.

### WAT Lipolysis

iWAT and eWAT were isolated from male mice and minced into 1 mm blocks in cold PBS. WAT explants were incubated at 37 °C for 2 h in KRH buffer (136 × 10^−3^ m NaCl, 4.7 × 10^−3^ m KCl, 1.25 × 10^−3^ m MgSO_4_, 1.25 × 10^−3^ m CaCl_2_, 20 × 10^−3^ m HEPES, pH 7.4, and 2% BSA), and then stimulated with isoproterenol (1 × 10^−6^ m). FFA and glycerol in KRH buffer were measured using NEFA‐HR reagents (Wako Chemicals, Richmond, VA) and Free Glycerol Reagents (Sigma, F6428), respectively.

### Energy Expenditure

Oxygen consumption (VO_2_), carbon dioxide production (VCO_2_), and food intake were measured at 20–23 °C and 12–12 h dark‐light cycles with free access to food and water using Comprehensive Laboratory Monitoring System (CLAMS, Columbus Instruments) in the University of Michigan Metabolic, Physiological and Behavioral Core. Carbohydrate and fatty acid oxidation were calculated following the formula: fat oxidation = 1.69 × VO_2_ – 1.69 × VCO_2_, glucose Oxidation = 4.57 × VCO_2_ − 3.23 × VO_2_.

### Cold Tolerance Tests

A mouse was placed in a precooled cage (with water but not food) and housed in a rodent environmental chamber (RIS33SD, Innovative Solutions, Beverly Hills, MI). Rectal temperature was measured hourly.

### Oil Red O Staining and Liver TAG

Liver frozen sections were prepared using a Leica cryostat (Leica Biosystems Nussloch GmbH, Nussloch, Germany) and stained with 1% Oil red O. Liver samples were homogenized in 1% acetic acid and extracted by chloroform:methanol (2:1). The organic phase was dried by evaporation and dissolved in isopropanol. TAG was completely hydrolyzed with ethanolic KOH, and glycerol was measured using a free glycerol reagent (Sigma, Free Glycerol Reagent F‐6428). TAG levels were calculated and normalized to liver weight.

### SVF and MEF Preparations

iWAT and eWAT were minced into 1 mm blocks in cold PBS containing CaCl_2_ (10 × 10^−3^ m) and incubated (shaking) at 37 °C for 20 min in PBS containing CaCl_2_ (10 × 10^−3^ m), collagenase D (1.5 units mL^−1^, Roche, 11 088 882 001), and dispase II (2.4 units mL^−1^, Roche, 4 942 078 001) as described before.^[^ ^1a^ ^]^ WAT suspension was filtered through a 100 µm filter and centrifuged at 200 *g* for 5 min. SVF cells (in pellets) were resuspended, washed in PBS containing CaCl_2_ (10 × 10^−3^ m) and fetal bovine serum (2%, FBS), and grown in DMEM containing glucose (25 × 10^−3^ m) and FBS (10%). Embryos (E17.5) were dissected in cold PBS, and internal organs and heads (used for genotyping) were removed. The remaining embryos (3–4 embryos per genotype) were minced in trypsin (1 mL) using a razor blade and incubated at 37 °C for 30 min. Digestion was stopped by adding DMEM (4 mL) supplemented with FBS (10%) and penicillin and streptomycin (50 units mL^−1^), and MEFs were dissociated by pipetting 10–20 times and grown in DMEM supplemented with FBS (10%).

### Adipocyte Differentiation and Lipolysis

3T3‐L1 preadipocytes were grown at 5% CO_2_ and 37 °C in DMEM containing glucose (25 × 10^−3^ m) and calf serum (8%). Confluent 3T3‐L1, SVF, and MEF cells were grown for additional 2 days, and then cultured for 2 days in a differentiation cocktail: DMEM (3T3‐L1) or DMEM/F12 (SVF and MEF) containing glucose (25 × 10^−3^ m), FBS (10%), insulin (0.1 × 10^−6^ m), dexamethasone (1 × 10^−6^ m, Sigma, D‐1756), 3‐isobutyl‐1‐methylxanthine (0.5 × 10^−3^ m, Sigma, I‐5879), indomethacin (0.125 × 10^−6^ m, Sigma, I‐7378), and rosiglitazone (0.1 × 10^−6^ m, Cayman Chemical Company, Ann Arbor, Michigan). The cells were grown for additional 2 days in DMEM (3T3‐L1) or DMEM/F12 (SVF, MEF) supplemented with FBS (10%) and insulin (0.1 × 10^−6^ m) and maintained in DMEM (3T3‐L1) or DMEM/F12 (SVF, MEF) supplemented with FBS (10%). Adipocytes were incubated in KRH buffer for 3 h and then stimulated with isoproterenol (1 × 10^−6^ m) for 3 h. FFA and glycerol in KRH buffer were measured using NEFA‐HR reagents and Free Glycerol Reagents, respectively.

### Human Tissues

De‐identified human visceral adipose specimens were obtained from the greater omentum with informed consent from obese nondiabetic subjects undergoing bariatric surgery. This was done under IRB‐approved protocols at the University of Michigan and the Ann Arbor VA Healthcare System under guidelines consistent with the 1964 Declaration of Helsinki and the 1974 Belmont Report. Human adipose tissue was digested with type II collagenase (2 mg mL^−1^ in PBS/2% BSA, Life Technologies Inc., Carlsbad, CA, USA), and SVF cells were isolated, plate‐adhered, and grown in DMEM supplemented with 10% FBS. Confluent SVF cells were grown for two additional days and then cultured for 14 days in a differentiation cocktail (DMEM/F12 supplemented 3‐isobutyl‐1‐methylxanthine (0.54 × 10^−3^ m), dexamethasone (0.1 × 10^−6^ m), insulin (500 × 10^−9^ m), ciglitazone (1 × 10^−6^ m), biotin (33 × 10^−6^ m), Transferrin (10 mg l^−1^), and *d*‐pantothenate (17 × 10^−6^ m)). Adipocytes were deprived for 4 h in DMEM/F12 (no phenol red) supplemented with BSA (0.1%, fatty acid free) and HEPES (10 × 10^−3^ m, pH 7.4), pretreated for 2 days with STM2457 (2.5 × 10^−6^ m, Cayman, 2499663‐01‐1), and then stimulated with isoproterenol (1 × 10^−6^ m) for 120 min. Glycerol and FFA in culture medium were measured using NEFA‐HR reagents and Free Glycerol Reagents, respectively. For tissue analyses, visceral adipose specimens of the obese group were from male patients with BMI of 30—58. Visceral fat specimens were obtained from cancer patients with BMI of 18—28 as lean control. The fat samples were collected within the past 8 years and stored at −80 °C.

### Immunoblotting

WAT was homogenized in a lysis buffer (Tris (50 × 10^−3^ m, pH 7.5), Nonidet P‐40 (1%), SDS(1%), NaCl (150 × 10^−3^ m), EGTA (2 × 10^−3^ m), Na_3_VO_4_ (1 × 10^−3^ m), NaF (100 × 10^−3^ m), Na_4_P_2_O_7_ (10 × 10^−3^ m), benzamidine (1 × 10^−3^ m), aprotinin (10 µg mL^−1^), leupeptin (10 µg mL^−1^), phenylmethylsulfonyl fluoride (1 × 10^−3^ m)) on ice using a glass Dounce homogenizer. Adipocyte cultures were lysed in the modified RIPA buffer. Livers were homogenized in the L‐RIPA lysis buffer (Tris (50 × 10^−3^ m, pH 7.5), Nonidet P‐40 (1%), SDS (1%), NaCl (150 × 10^−3^ m), EGTA (2 × 10^−3^ m), Na_3_VO_4_ (1 × 10^−3^ m), NaF (100 × 10^−3^ m), Na_4_P_2_O_7_ (10 × 10^−3^ m), benzamidine (1 × 10^−3^ m), aprotinin (10 µg mL^−1^), leupeptin (10 µg mL^−1^), phenylmethylsulfonyl fluoride (1 × 10^−3^ m)) using TissueLyser II (QIAGEN, Valencia, CA). Proteins were separated by SDS‐PAGE and immunoblotted with the indicated antibodies (Table S1, Supporting Information)

### m6A Dot Blot Assay and m6A RIP Assay

For dot blot assays, total RNA (2 µg) was spotted on N^+^ membrane, UV‐crosslinked using the auto‐crosslink programs by a XL‐1500 UV crosslinker (Spectrolinker, Westbury, New York), and immunoblotted with anti‐m6A antibody. The blots were stained with methylene blue to visualize total RNA. For m6A RIP assays, mouse or human WAT, or adipocyte cultures were lysed on ice for 20 min in NETN buffer: Tris‐Cl (20 × 10^−3^ m, pH 8.0), NaCl (100 × 10^−3^ m), EDTA (1 × 10^−3^ m), NP‐40 (0.5%), Na_3_VO_4_ (1 × 10^−3^ m), NaF (100 × 10^−3^ m), Na_4_P_2_O_7_ (10 × 10^−3^ m), benzamidine (1 × 10^−3^ m), aprotinin (10 µg mL^−1^), leupeptin (10 µg mL^−1^), phenylmethylsulfonyl fluoride (1 × 10^−3^ m), and RNasin (40 U mL^−1^ Promega, N2511). Lysates were centrifugated at 13 000 rpm for 10 min at 4 °C. Supernatants were incubated at 4 °C with 1 µL anti‐m6A antibody for 2 h and then with protein‐A agarose beads (20 µL) for an additional 1 h (with rotation). The beads were washed with the NETN buffer and their RNA was extracted using Trizol reagent (ThermoFisher, 15 596 026) and used for qPCR. Primers were listed in Table S2 (Supporting Information).

### Ribosome Profiling

Adipocytes were washed with ice‐cold PBS supplemented with cycloheximide (100 µg mL^−1^), scraped off plates, and collected by centrifugation at 1000 ×g for 5 min at 4 °C, and lysed in a lysis buffer: KCl (50 × 10^−3^ m), Tris·HCl (20 × 10^−3^ m, pH 7.4), MgCl_2_ (10 × 10^−3^ m), Triton X‐100 (1%), 1,4‐dithiothreitol (1 × 10^−3^ m), sodium deoxycholate (0.5% w/v), cycloheximide (100 µg mL^−1^), Na_4_P_2_O_7_ (10 × 10^−3^ m), benzamidine (1 × 10^−3^ m), aprotinin (10 µg mL^−1^), leupeptin (10 µg mL^−1^), phenylmethylsulfonyl fluoride (1 × 10^−3^ m), and RNasin. Cell extracts were centrifuged twice at 2000 × *g* for 5 min and at 13000 × *g* for 5 min. A 7%−47% (w/v) sucrose gradient solution, containing KCl (50 × 10^−3^ m), MgCl_2_ (10 × 10^−3^ m), Tris·HCl (20 × 10^−3^ m, pH7.4) was prepared in an ultracentrifugation tube (Backman, 342 413) using a Gradient Maker (C.B.S. Scientific, GM‐20). Cell extracts (6 mL) were loaded on the top of sucrose gradient solution (6 mL) and ultracentrifuged at 260808 × *g* (39 000 rpm, Beckman SW 41 rotor) for 90 min in the slow acceleration mode. Consecutive fractions (200 µL per fraction) were collected (total 60 fractions), and their optical absorbance (260 nm) was measured by Synergy 2 microplate reader (BioTek Instruments) to verify monosome and polysome separation. Fractions 1–36 were combined and defined as the monosome pool while fractions 37–60 were combined as the polysome pool. Total RNA was extracted from the two pools to measure individual transcript abundance by qPCR, and polysome‐bound/monosome‐bound mRNA ratios were calculated.

### OPP Pulldown Assays

This work followed a previously described method.^[^ ^21^ ^]^ Briefly, adipocytes were incubated for 3 h at 37 °C with OPP (30 × 10^−6^ m) in growth medium, washed with cold PBS, and lysed in a RIPA buffer. OPP‐tagged nascent proteins in cell extracts were conjugated with biotin using biotin picolyl azide and a Click‐&‐Go protein reaction buffer Kit (Fisher, NC1438136) according to the manufacturer’ instructions. The extracts were precipitated with five volumes of cold acetone overnight at −20 °C and centrifuged at 4000 × *g* at 4 °C for 10 min. The pellets were wash with cold methanol and resuspend in PBS containing SDS (1%). The suspension (500 µg proteins) was incubated with streptavidin magnetic beads (Fisher, 50‐210‐8415) at 4 °C overnight with gentle rotation. The beads were wash with cold PBS containing NP40 (1%) and SDS (0.1%) and boiled in 2× Laemmli sample buffer for 10 min to elute OPP‐tagged nascent proteins. OPP‐tagged proteins were quantified by immunoblotting using the indicated antibodies.

### RNA Decay Assays

Adipocyte cultures were treated with actinomycin D (1 × 10^−6^ m, transcription inhibitor) for 0–6 h. Total RNA was extracted to measure mRNA abundance of individual transcripts by qPCR (normalized to 18S levels).

### RNA‐Seq and Data Analysis

*Mettl14^f/f^* and *Mettl14^Δfat^* male mice (8 weeks) were fed a HFD for 16 weeks and eWAT was dissected. Total RNA was extracted by RNeasy Mini Kit (Qiagen Cat#74 104) and used for RNA‐seq (BGI Genomics, Hong Kong, China). STAR, HTSeq, and DESeq2 software were used to analyze differentially expressed genes between *Mettl14^f/f^* and *Mettl14^Δfat^* eWAT. The RNA‐seq data were deposited in the Gene Expression Omnibus (GEO, GSE233782).

### qPCR

Total RNAs were extracted using TRIzol reagents (Life technologies). Relative mRNA abundance was measured using Radiant SYBR Green 2× Lo‐ROX qPCR Kits (Alkali Scientific, Pompano Beach, FL), 2× Universal SYBR green fast qPCR mix (ABclonal Technology, Cat# RK21203), Mastercycler qPCR system (Eppendorf), and StepOnePlus RT PCR Systems (Life Technologies Corporation, NY, USA). Primers were listed in Table S2 (Supporting Information).

### Statistical Analysis

Data were presented as means ± SEM. Differences between two groups were analyzed by two‐tailed Student's *t* test. Comparisons between more than two groups/variables were analyzed by one‐way ANOVA/Tukey's post hoc test using GraphPad Prism 8. A *p* value less than 0.05 was considered significant.

## Conflict of Interest

The authors declare no conflict of interest.

## Author Contributions

Q.K. and X.Z. conducted the experiments, A.K. and R.O. provided human SVFs, D.R. provided *Mettl14^f/f^* mice, O.M. provided *adiponectin‐CreERT* mice, Q.K. and L.R. designed the experiments and wrote the paper, and Q.K., X.Z., D.R., O.M., R.O., and L.R. edited the paper.

## Supporting information

Supporting Information

## Data Availability

The data that support the findings of this study are available in the supplementary material of this article.
