# Supplementary material for: Adipose METTL14‐Elicited N6‐Methyladenosine Promotes Obesity, Insulin Resistance, and NAFLD Through Suppressing β Adrenergic Signaling and Lipolysis
Source: Adv Sci (Weinh). 2023 Aug 1;10(28):2301645. doi: 10.1002/advs.202301645 (PMC10558699; doi:10.1002/advs.202301645)
Supplement: Supplementary file 1 — Supporting Information [file ADVS-10-2301645-s001.pdf]

ADVANCED SCIENCE
Open Access

Supporting Information

for *Adv. Sci.*, DOI 10.1002/advs.202301645

Adipose METTL14-Elicited N^6-Methyladenosine Promotes Obesity, Insulin Resistance, and NAFLD Through Suppressing β Adrenergic Signaling and Lipolysis

*Qianqian Kang, Xiaorong Zhu, Decheng Ren, Alexander Ky, Ormond A. MacDougald, Robert W. O'Rourke and Liangyou Rui**

Supporting Information

Adipose Mettl14-elicited N^6-methyladenosine promotes obesity, insulin resistance, and NAFLD through suppressing β adrenergic signaling lipolysis

*Qianqian Kang, Xiaorong Zhu, Decheng Ren, Alexander Ky, Ormond MacDougald, Robert W. O'Rourke, Liangyou Rui**

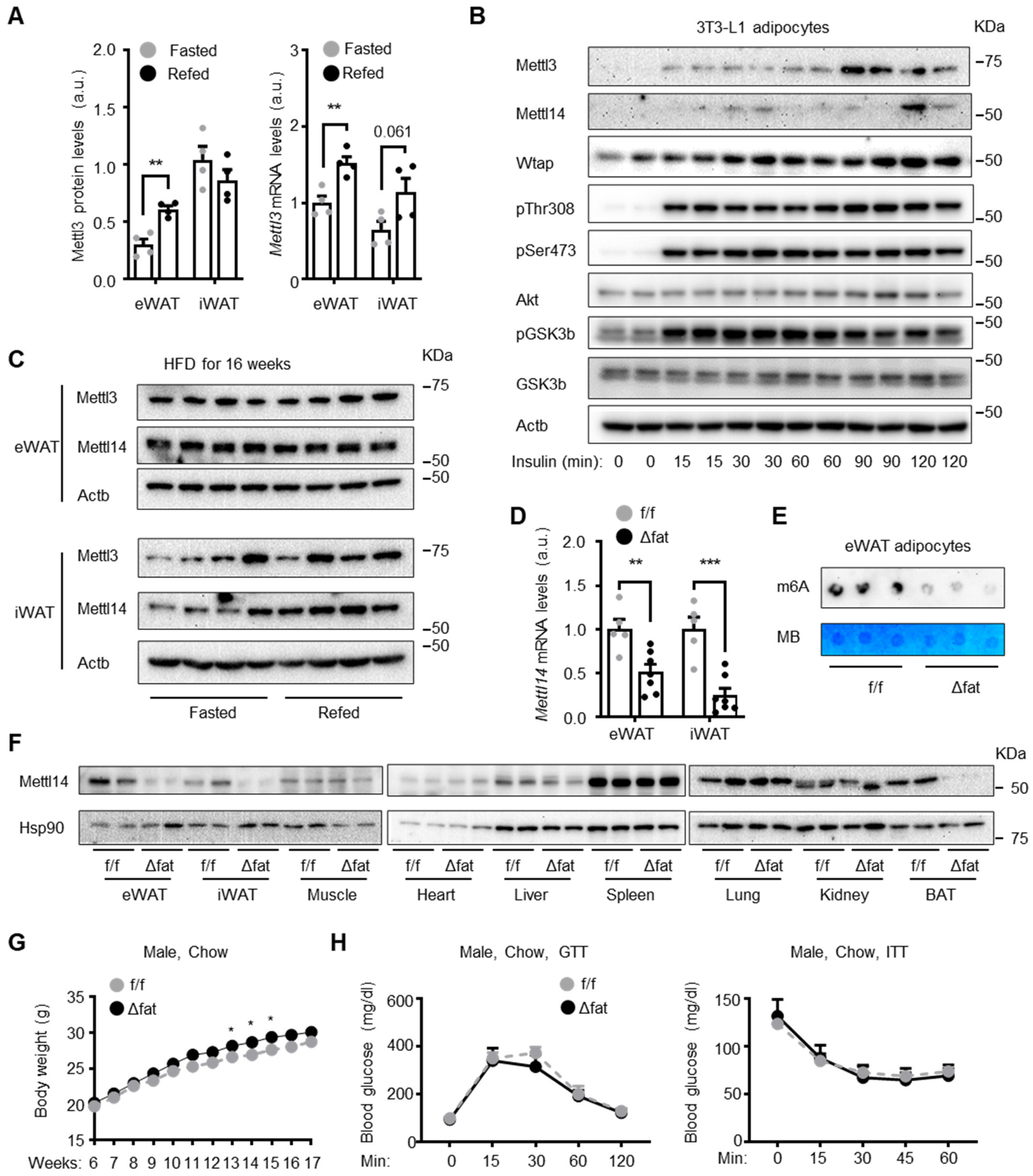

Figure S1. *Mettl14*$^{\Delta fat}$ mice are normal on chow diet. A) C57BL/6J male mice (8 weeks old) were fasted overnight and then refed for 3 h. Mettl3 protein (normalized to Actb) and mRNA (normalized to 36B4) levels were measured in eWAT and iWAT by immunoblotting and qPCR, respectively (n=4 mice per group), as described in Figure 1A. a.u.: arbitrary unit. **B)** 3T3-L1 cells were differentiated into adipocytes and stimulated with 5 µg/ml insulin for the indicated durations. Cell extracts were immunoblotted with the indicated antibodies. **C)** C57BL/6J male mice (8 weeks old) were fed a HFD for 16 weeks and then fasted overnight and refed for 3 h. WAT extracts were immunoblotted with the indicated antibodies. **D)** *Mettl14*

mRNA levels were measured in iWAT and eWAT (24 weeks old) by qPCR and normalized to 36B4 levels. a.u.: arbitrary unit. *Mettl14*$^{f/f}$: n=5, *Mettl14*$^{\Delta fat}$: n=7. **E)** Primary adipocytes were isolated from eWAT, and total RNA was extracted for m6A dot blot assays. **F)** Tissues were harvested from *Mettl14*$^{f/f}$ and *Mettl14*$^{\Delta fat}$ male mice at 24 weeks of age. Tissue extracts were immunoblotted with antibodies to Mettl14 and Hsp90. **G-H)** *Mettl14*$^{f/f}$ and *Mettl14*$^{\Delta fat}$ male mice were placed on chow diet. **G)** Growth curves. *Mettl14*$^{f/f}$: n=7, *Mettl14*$^{\Delta fat}$: n=5. **H)** GTT and ITT at 18 weeks of age. *Mettl14*$^{f/f}$: n=7, *Mettl14*$^{\Delta fat}$: n=5. Data are presented as mean ± SEM. *p<0.05, **p<0.01, ***p<0.001, Student's *t* test.

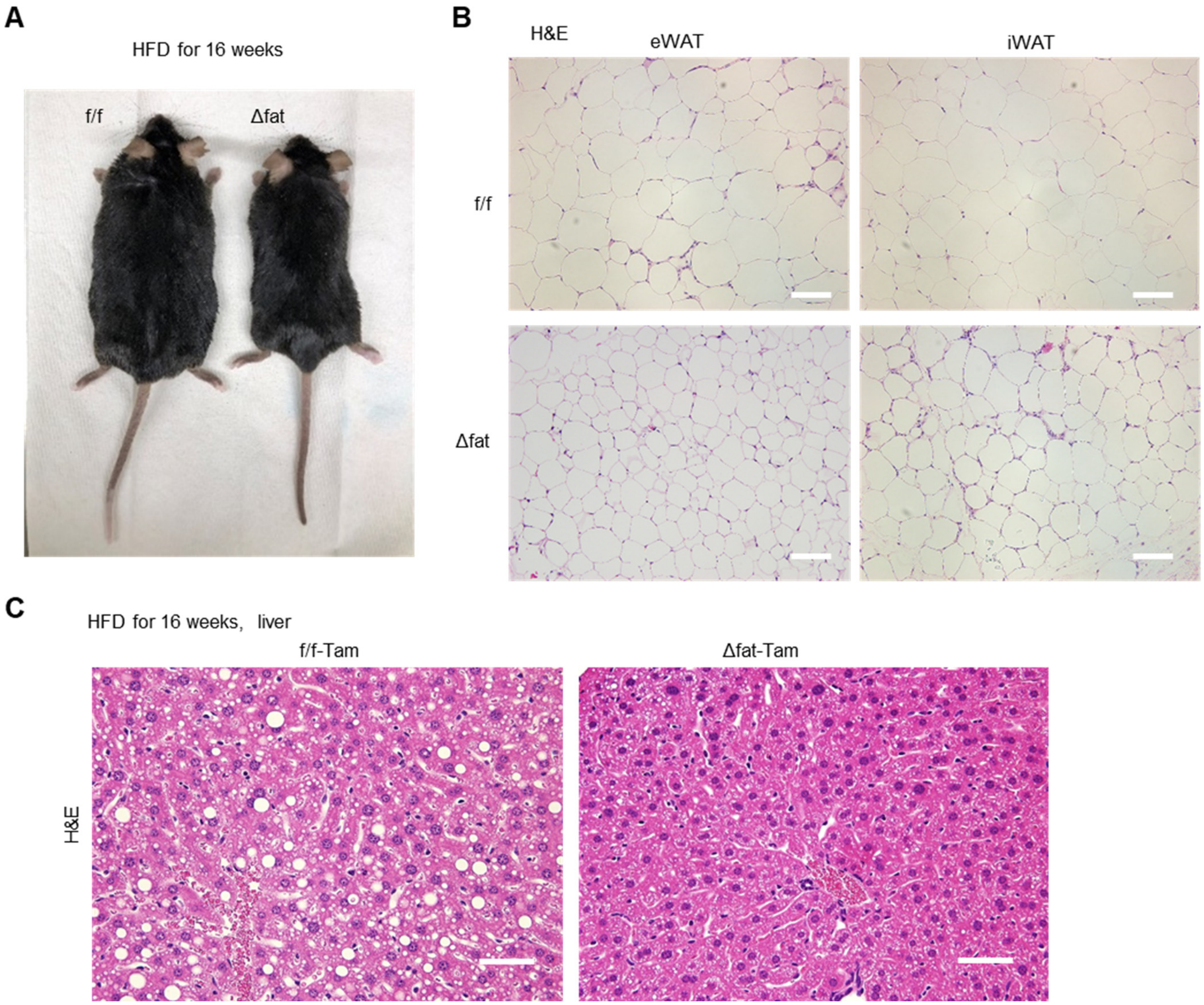

Figure S2. *Mettl14*$^{\Delta fat}$ mice are resistant to HFD-induced obesity and NAFLD. A-B) *Mettl14*$^{f/f}$ and *Mettl14*$^{\Delta fat}$ male mice (8 weeks) were fed a HFD for 16 weeks. **A)** Mouse images. **B)** H&E staining of iWAT and eWAT sections. Scale bar: 200 μm. **C)** H&E staining of liver sections. Scale bar: 50 μm. *Mettl14*$^{f/f\text{-}Tam}$ and *Mettl14*$^{\Delta fat\text{-}Tam}$ male mice (7 weeks) were injected with tamoxifen and one week later, they were fed a HFD for 10 weeks.

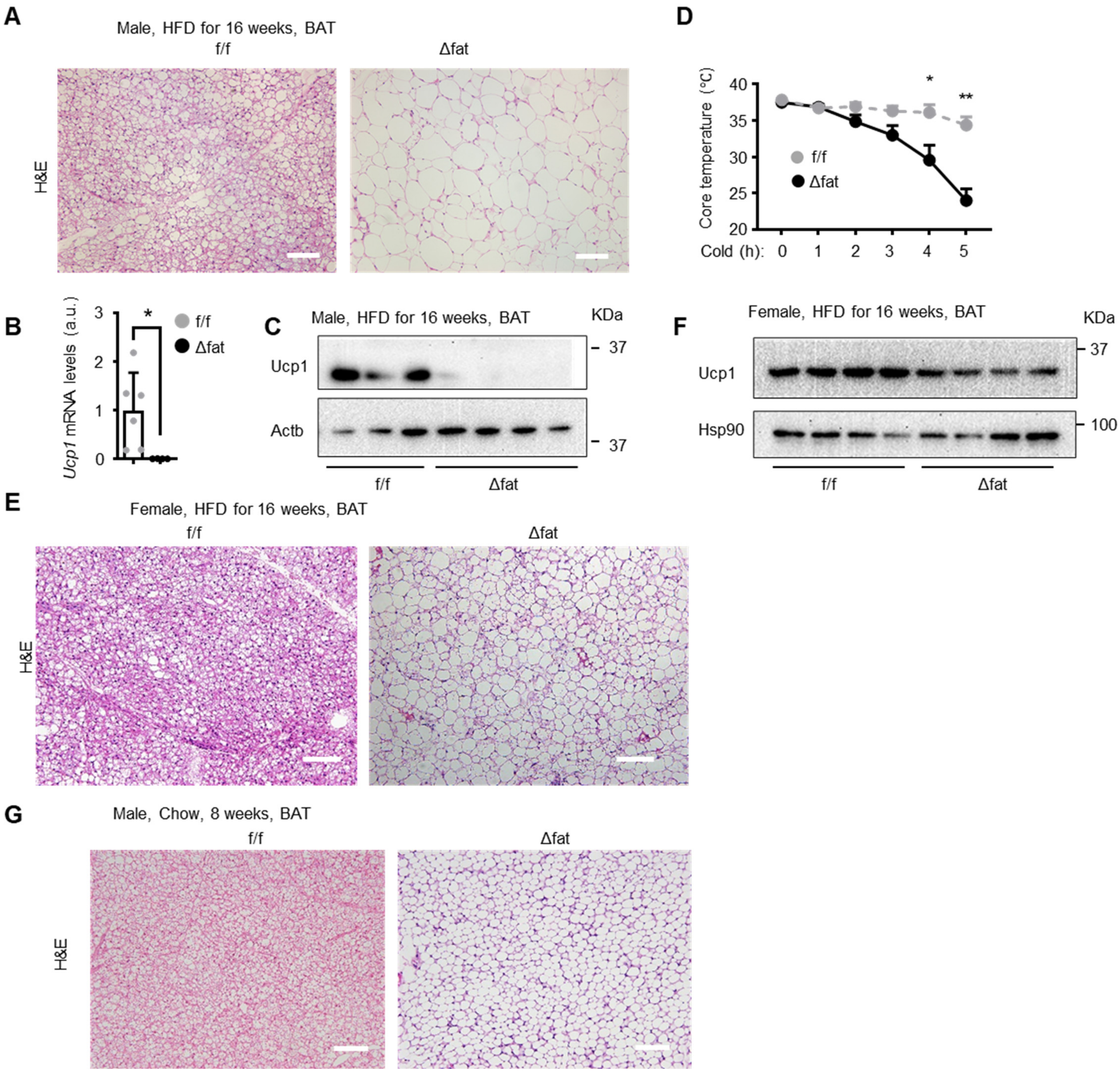

Figure S3. *Mettl14$^{\Delta fat}$* mice display BAT whitening and cold intolerance. A-D) *Mettl14$^{f/f}$* and *Mettl14$^{\Delta fat}$* male mice (8 weeks) were fed a HFD for 16 weeks. **A)** H&E staining of BAT sections. Scale bar: 200 µm. **B)** *Ucp1* mRNA levels (normalized to 36B4 levels). a.u.: arbitrary unit. *Mettl14$^{f/f}$*: n=6, *Mettl14$^{\Delta fat}$*: n=4. **C)** BAT extracts were immunoblotted with antibodies to Ucp1 and Actb. **(D)** *Mettl14$^{f/f}$* and *Mettl14$^{\Delta fat}$* males (HFD for 12 weeks) were fasted overnight and exposed to 4^{0}C cold temperature for 5 h in the absence of food. Rectal temperatures were monitored. *Mettl14$^{f/f}$*: n=4, *Mettl14$^{\Delta fat}$*: n=4. **E-F)** *Mettl14$^{f/f}$* and *Mettl14$^{\Delta fat}$* female mice (8 weeks) were fed a HFD for 16 weeks. **E)** H&E staining of BAT sections. **F)** BAT extracts were immunoblotted with antibodies to Ucp1 and Hsp90. **G)** H&E staining of BAT sections at 8 weeks of age on chow diet. Data are presented as mean ± SEM. *p<0.05, **p<0.01, ***p<0.001, Student's *t* test (**B**) and 1-way ANOVA (**D**).

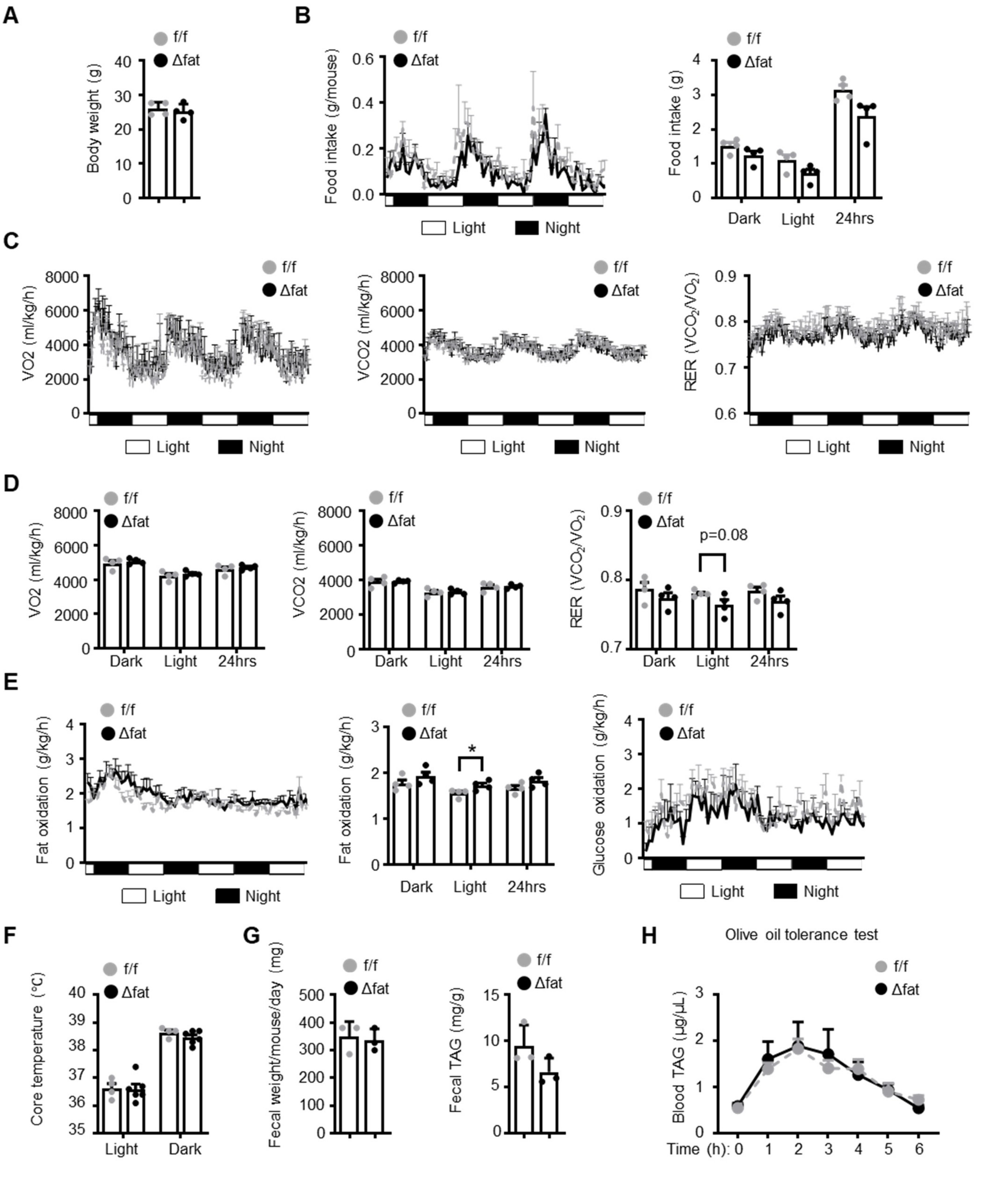

Figure S4. *Mettl14$^{\Delta fat}$* mice display normal food intake and energy expenditure. A-E) *Mettl14$^{f/f}$* and *Mettl14$^{\Delta fat}$* males (8 weeks) were fed a HFD for one week and subjected CLAMS tests. **A)** Body weight. *Mettl14$^{f/f}$*: n=4, *Mettl14$^{\Delta fat}$*: n=4. **B)** Food intake. Left: sampled at a 1 h-interval, right: average food intake in the dark, light, and dark/light phases. *Mettl14$^{f/f}$*: n=4, *Mettl14$^{\Delta fat}$*: n=4. **C)** O_2 consumption, CO_2 production (normalized to lean body mass), and respiratory exchange ratio (RER) was sampled at a 24-min interval. Calculated energy expenditure (EE) plots over time. *Mettl14$^{f/f}$*: n=4, *Mettl14$^{\Delta fat}$*: n=4. **D)** Average VO_2, VCO_2

and energy expenditure (normalized to lean mass). *Mettl14*$^{f/f}$: n=4, *Mettl14*$^{\Delta fat}$: n=4. **E)** Fatty acid oxidation and glucose oxidation rates (sampled at a 24-min interval). **(F-H)** Male mice (8 weeks) were fed a HFD for 6 weeks. **F)** Body core temperate. *Mettl14*$^{f/f}$: n=4, *Mettl14*$^{\Delta fat}$: n=6. **G)** Feces weight and fecal TAG levels (normalized to feces weight, n=3 mice per group). **(H)** Mice ere fasted overnight and administrated with olive oil (5 ml/kg body weight, via oral gavage). Plasma TAG levels monitored post gavage. *Mettl14*$^{f/f}$: n=4, *Mettl14*$^{\Delta fat}$: n=6. Data are presented as mean ± SEM. *p<0.05, **p<0.01, ***p<0.001, Student's *t* test.

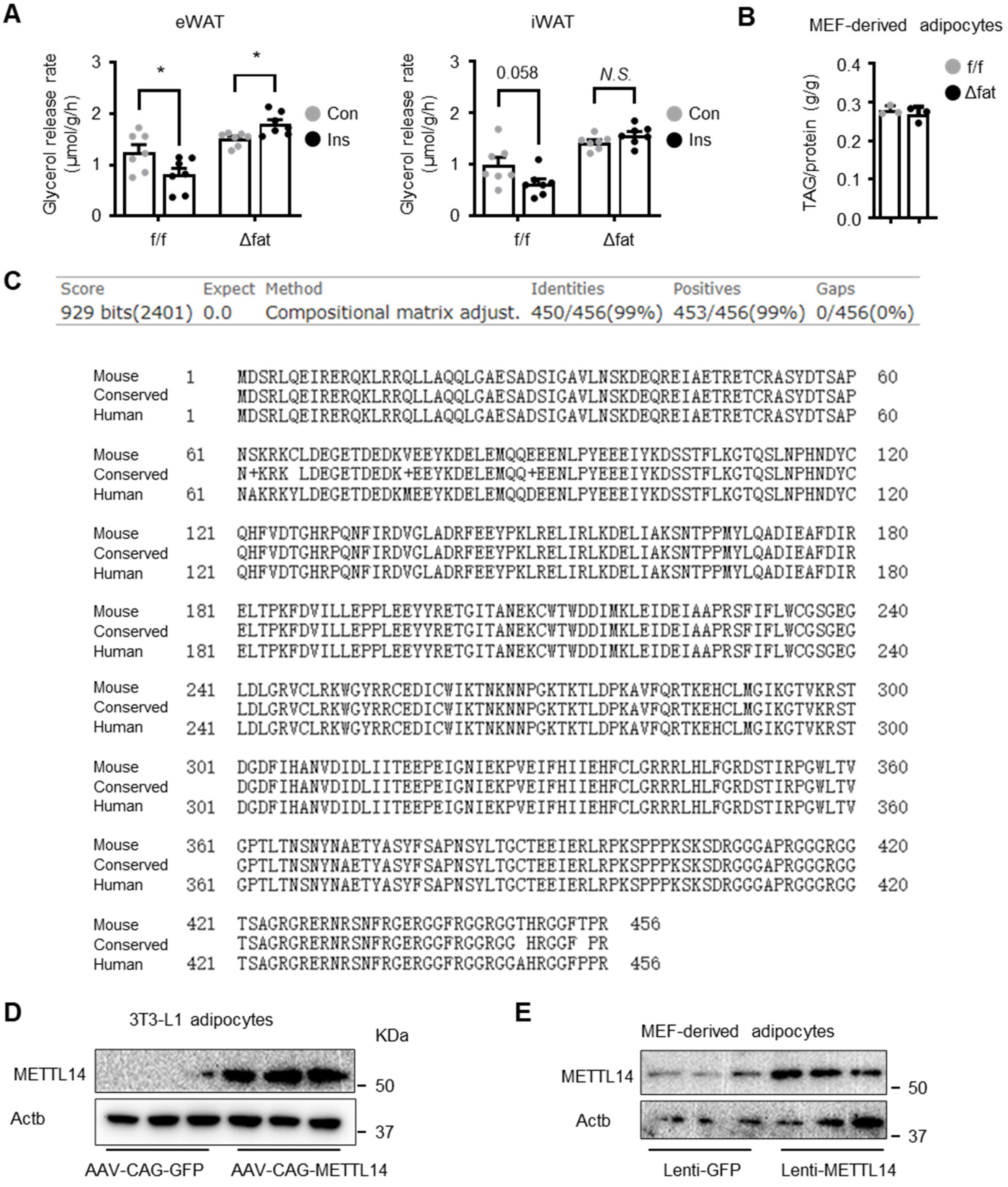

Figure S5. Mettl14 cell-autonomously suppresses adipose lipolysis. A) WAT was isolated from male mice (8 weeks) and stimulated with isoproterenol (1 μM) for 3 h in the presence or absence of insulin (100 nM). Glycerol secretion rates were measured and normalized to WAT weights. **B)** MEFs were differentiated into adipocytes for 8 days using an adipose differentiation cocktail. Total TAG levels were measured and normalized to proteins (n=3 repeats per group). **C)** Comparation of mouse and human METTL14 amino acid sequences. **D)** 3T3-L1 cells were differentiated into adipocytes and transduced with AAV-CAG-METTL14 or AAV-CAG-GFP vectors (for 3 days). Cell lysates were immunoblotted with antibodies to METTL14 and Actb. **E)** MEFs were transduced with METTL14 or GFP lentiviral vectors and differentiated into adipocytes. Cell lysates were immunoblotted with antibodies to METTL14 and Actb. Data are presented as mean ± SEM.

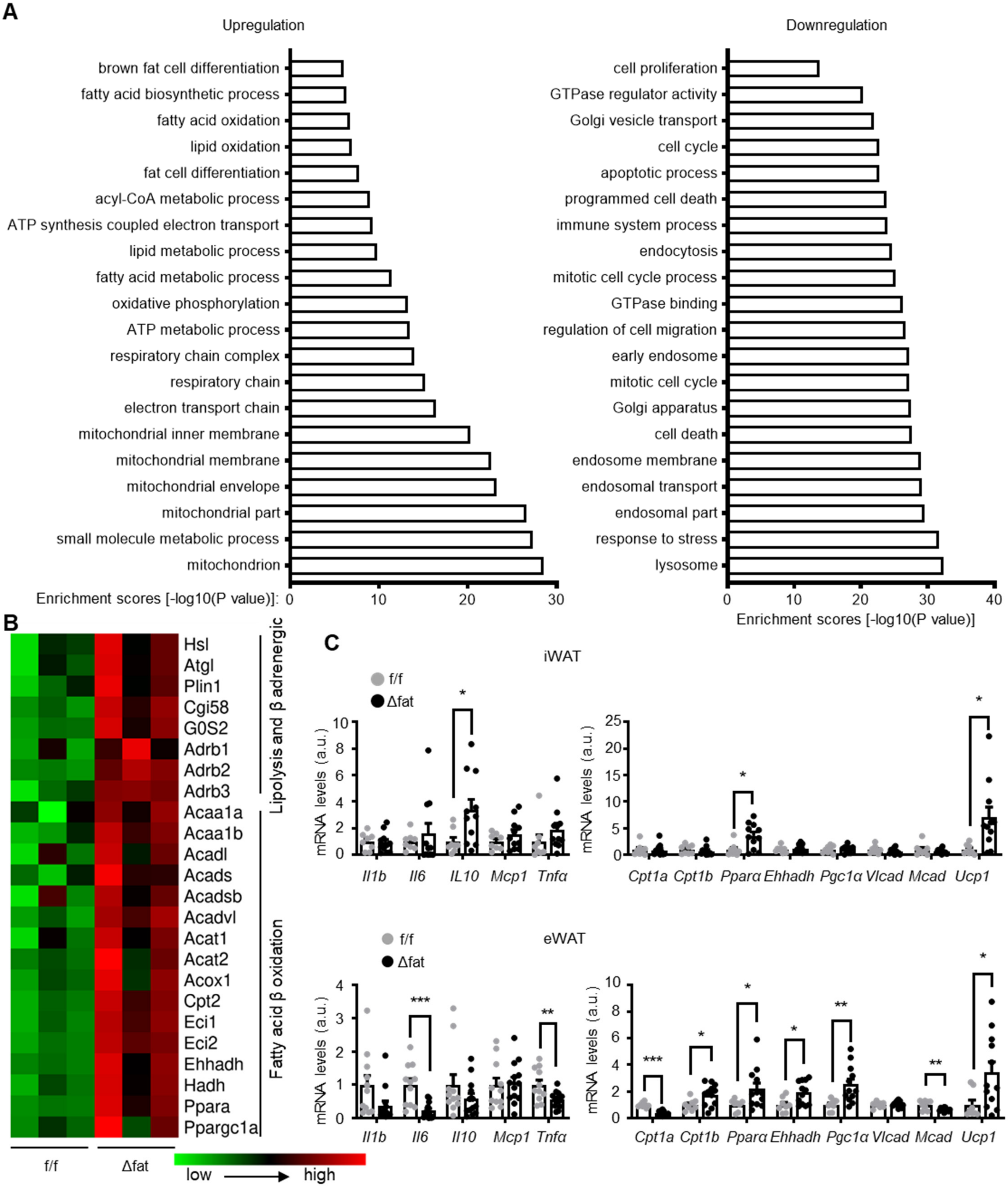

Figure S6. Adipose Mettl14 regulates the levels of lipolysis regulators and β-adrenergic signaling in adipocytes. *Mettl14$^{f/f}$* and *Mettl14$^{\Delta fat}$* males (8 weeks old) were fed a HFD for 16 weeks. **A-B)** eWAT was harvested for RNA-seq (n = 3). **A)** GO analyses of upregulated and downregulated genes. **B)** Gene

expression heatmap. **(C)** iWAT and eWAT gene expression was measured by qPCR and normalized to 36B4 levels. a.u.: arbitrary unit. iWAT *Mettl14*$^{f/f}$: n=8, iWAT *Mettl14*$^{\Delta fat}$: n=11, eWAT *Mettl14*$^{f/f}$: n=8-11, eWAT *Mettl14*$^{\Delta fat}$: n=11-13. Data are presented as mean ± SEM. *p<0.05, **p<0.01, ***p<0.001, Student's *t* test.

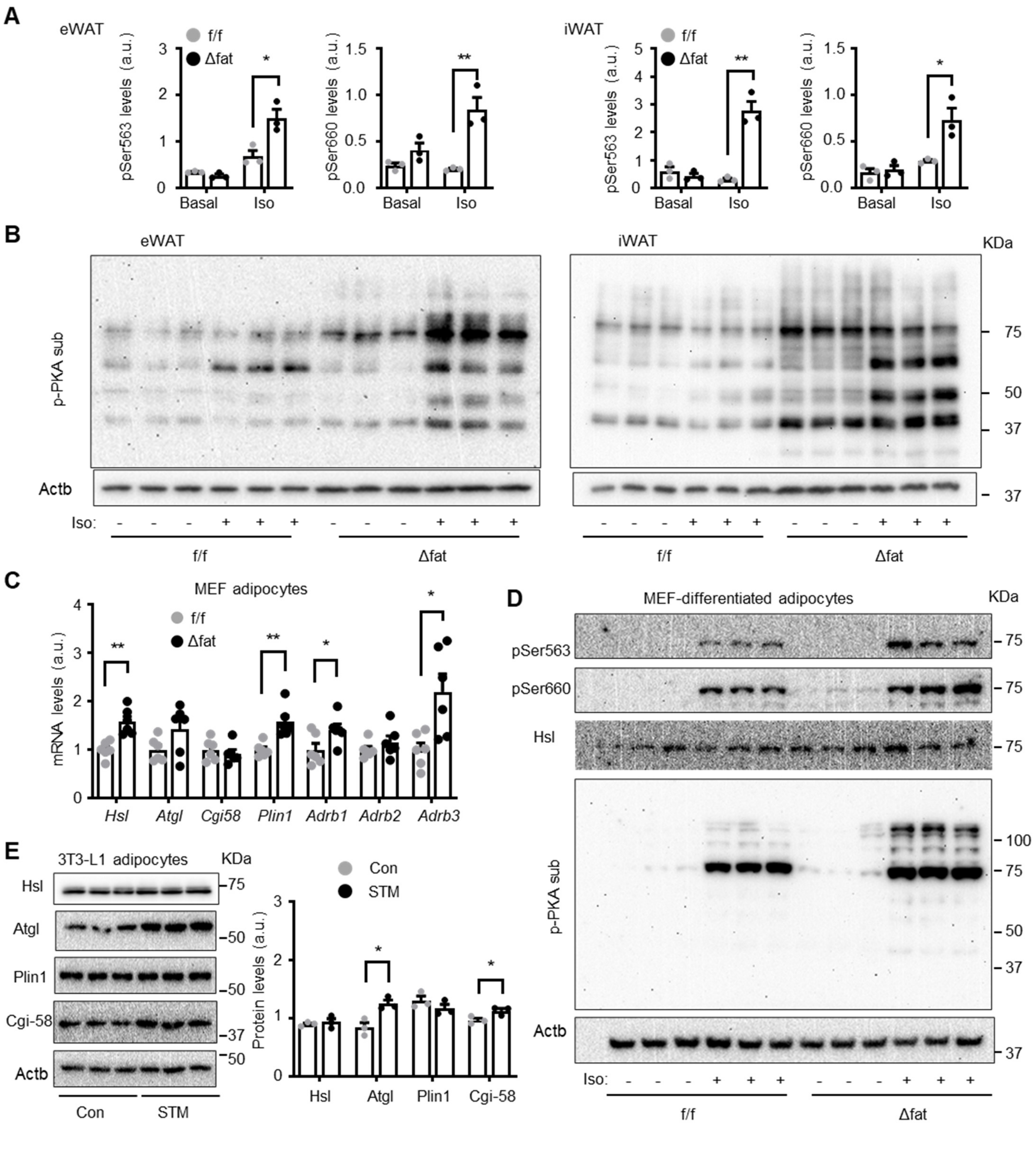

Figure S7. Adipose Mettl14 regulates the levels of lipolysis regulators and β-adrenergic signaling in adipocytes. A-B) Male mice (8 weeks) were fed a HFD for 4 weeks. iWAT and eWAT were isolated and stimulated with isoproterenol (1 μM) for 15 min. **A)** WAT extracts were immunoblotted with antibodies to phospho-HSL (pSer563, pSer660), and pSer563 and pSer660 levels were quantified and normalized to total Hsl levels (n=3 mice per group). a.u.: arbitrary unit. **B)** WAT extracts were immunoblotted with antibodies to pan-phospho-PKA substrates and Actb. **C)** MEF cells were differentiated into adipocytes for 8 days. mRNA levels were measured by qPCR and normalized to 36B4 levels (n=6 repeats per group). **D)** MEFs were differentiated into adipocytes in vitro for 6 days, serum-deprived for 8 h, and stimulated with isoproterenol (1 μM) for 15 min. Cell extracts were immunoblotted with the indicated antibodies. **E)** 3T3-L1 cells were differentiated into adipocytes and treated with STM2457 (5 μM) for 48 h. Cell extracts were immunoblotted with the indicated antibodies. Protein levels were quantified and normalized to Actb levels (n=3 repeats per group). Data are presented as mean ± SEM. *p<0.05, **p<0.01, Student's *t* test.

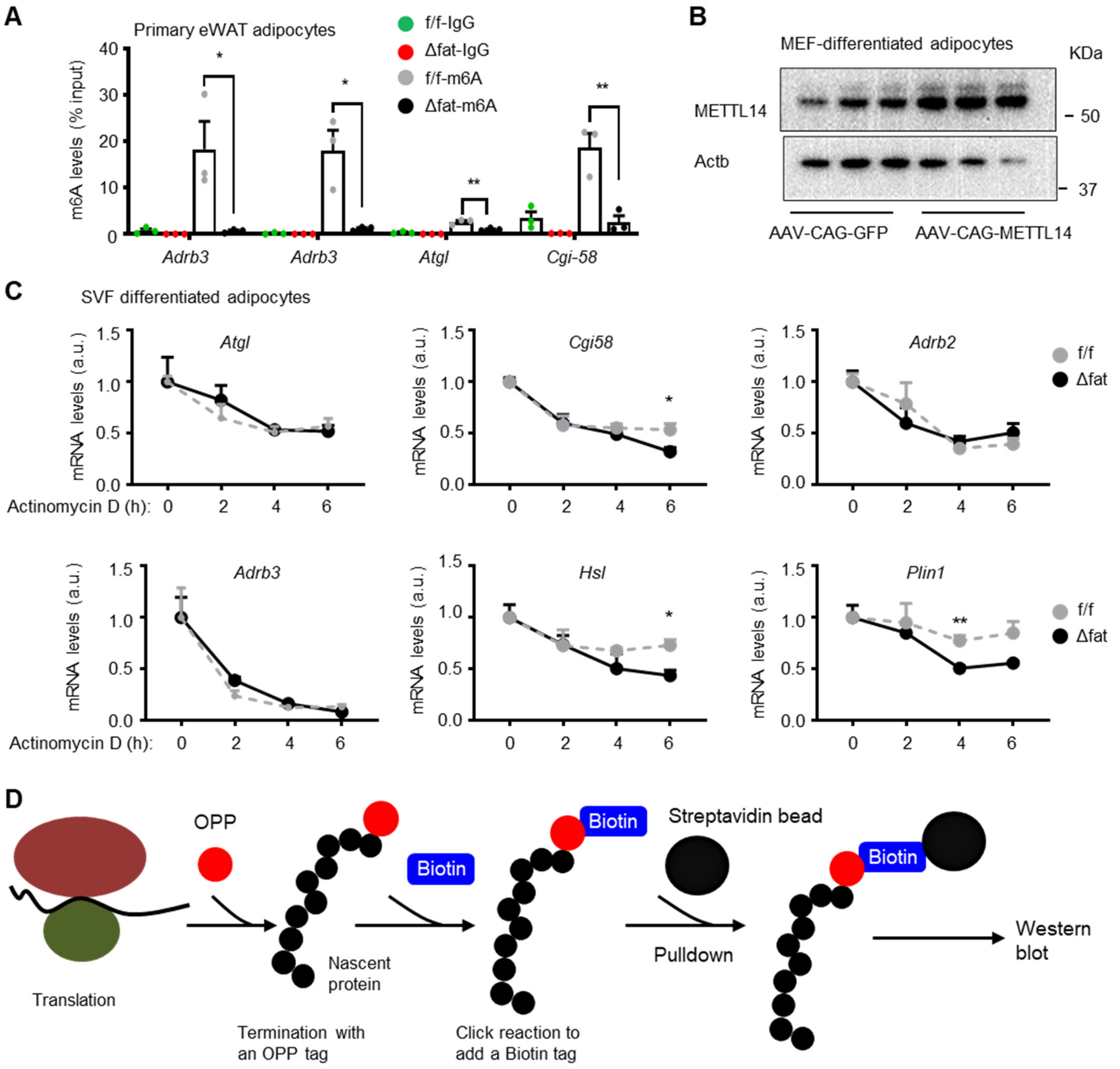

Figure S8. Influences of Mettl14-mediated m6A modification on stability and translational efficiency of lipolysis-related transcripts. A) Primary adipocytes were isolated from eWAT (8 weeks old) to extract total RNA. RNA was immunoprecipitated with anti-m6A antibody (a copy of the results of Figure 6A) or IgG. Precipitated RNA was extracted and used to measure the indicated transcripts by qPCR (normalized to inputs, n=3 mice per group). **B)** MEF-derived adipocytes were transduced with AAV vectors, and cell extracts were immunoblotted with the indicated antibodies. **C)** SVF cells were isolated from iWAT (8 weeks old) and differentiated into adipocytes. Adipocytes were treated with actinomycin D (1 μM) for 0-6 h. Total RNA was extracted to measure mRNA abundance by qPCR (normalized to 18S levels). The results were presented as percentages of baseline values (n=3 repeats per group). a.u.: arbitrary unit. **D)** Schematic representation of OPP pulldown assays. Data are presented as mean ± SEM. *$p<0.05$, **$p<0.01$, ANOVA.

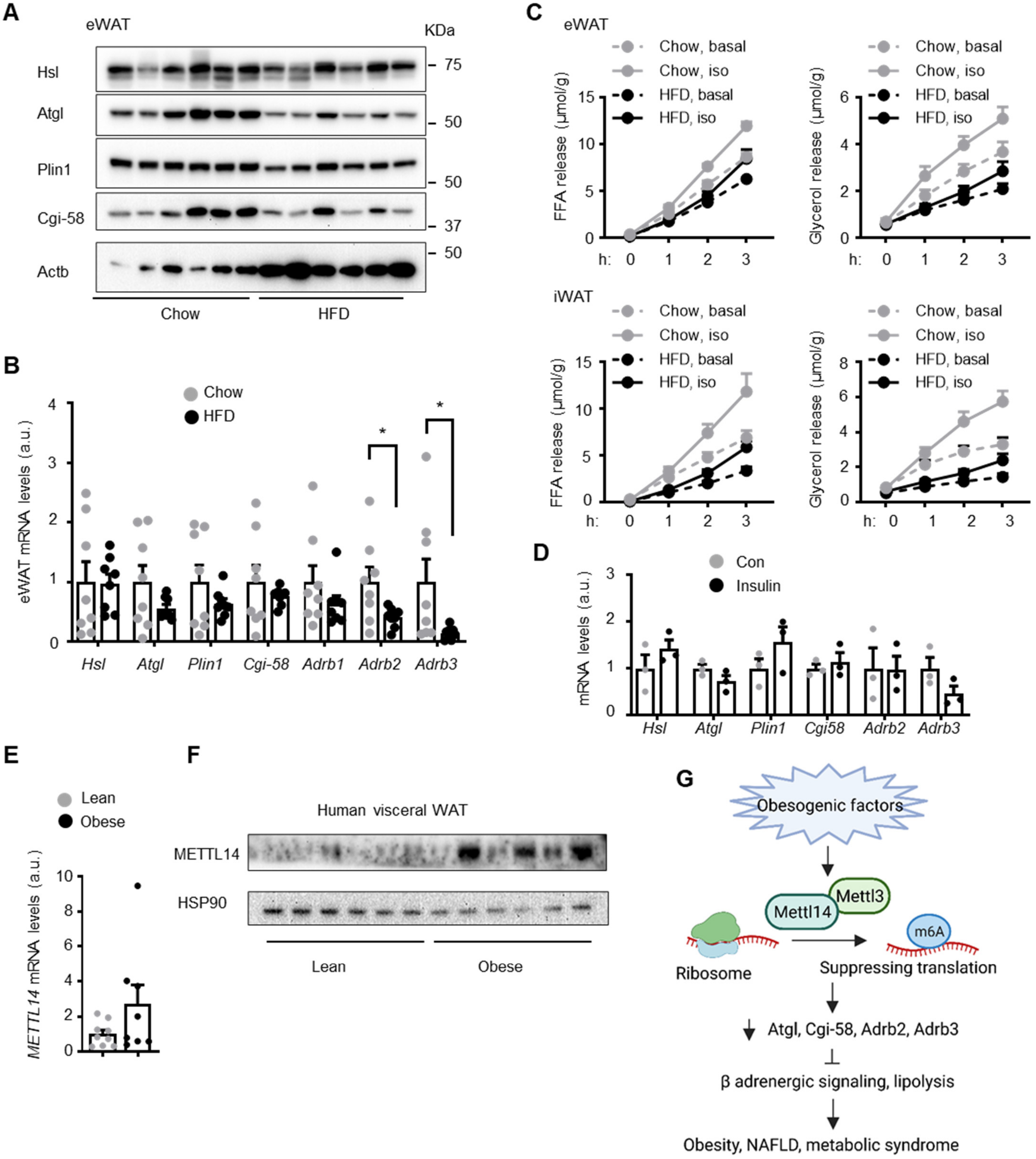

Figure S9. Influences of Mettl14-mediated m6A modification on stability and translational efficiency of lipolysis-related transcripts. C57BL/6J male mice (8 weeks old) were fed a HFD for 10 weeks. **A)** eWAT extracts were immunoblotted with the indicated antibodies. **B)** eWAT mRNA levels were measured by qPCR (normalized to 36B4 levels, n=8 mice per group). **C)** iWAT and eWAT explants were isolated and stimulated with isoproterenol (1 μM, Iso) for 3 h. Glycerol- and FFA-releasing rates were measured and normalized to WAT weight. a.u.: arbitrary unit. Chow: n=7, HFD: n=6. **D)** 3T3-L1 cells were differentiated into adipocytes and stimulated with 5 μg/ml insulin for 16 h. Gene expression was measured by qPCR and

normalized to 36B4 levels (n=4 repeats per group). **E-F)** Human visceral WAT samples were used to extract total RNA or prepare WAT extracts. **E)** Gene expression was measured by qPCR (normalized to 18S). Lean: n=9, obese: n=8. **F)** WAT extracts were immunoblotted with anti-METTL14 antibody. **G)** Obesogenic factors upregulate adipose METTL3/ and METTL14 that in turn install m6A in transcripts encoding β adrenergic (Adrb2/3) and lipolytic (Atgl/Cgi-58) pathway mediators. m6A methylation influences processing and suppresses translation of target mRNAs, thereby suppressing β adrenergic signaling (catecholamine resistance) and lipolysis to increase adipose expansion. Thus, adipose m6A-centric epitranscriptomic reprogramming promotes obesity, NAFLD, and metabolic disorders. Data are presented as mean ± SEM. *p<0.05, **p<0.01, ***p<0.001, Student's *t* test.

ANTIBODY	SOURCE	Cat#	Blot
METTL3	Proteintech Group	15073-1-AP	1:1000
METTL14	Sigma	HPA038002	1:2000
m6A	Cell Signaling Technology	56593	1:1000
Actb	Abclonal	AC026	1:20000
pAkt (pThr308)	Cell Signaling Technology	2965	1:1000
pAkt (pSer473)	Cell Signaling Technology	4060	1:1000
Akt	Cell Signaling Technology	2920	1:1000
pGSK3b	Cell Signaling Technology	5558	1:1000
GSK3b	Cell Signaling Technology	9315	1:1000
WTAP	Proteintech Group	60188-1-Ig	1:1000
HSP90	Cell Signaling Technology	4877	1:2000
pHSL (pSer563)	Cell Signaling Technology	4139	1:1000
pHSL (pSer660)	Cell Signaling Technology	4126	1:1000
HSL	Cell Signaling Technology	18381	1:1000
Plin1	Cell Signaling Technology	9349	1:2000
CGI-58	Proteintech Group	12201-1-AP	1:2000
pPKA sub	Cell Signaling Technology	9624	1:1000
ATGL	Cell Signaling Technology	2439	1:2000
Adrb2	Abclonal	A2048	1:1000
Adrb3	Santa Cruz	sc-515763	1:500
UCP1	Cell Signaling Technology	14670	1:5000

Table S1. Antibody list

Genes	Forward	Reverse
Mettl3	AGCAGGACTCTGGGCACTT	GCTTAGGGCCGCTAGAGGTA
36B4	AAGCGCGTCCTGGCATTGTCT	CCGCAGGGGCAGCAGTGGT
Mettl14	GCTTGCGAAAGTGGGGTTAC	AATGAAGTCCCCGTCTGTGC
Hsl	ACGCTACACAAAGGCTGCTT	TCGTTGCGTTTGTAGTGCTC
Atgl	TTCACCATCCGCTTGTTGGAG	AGATGGTCACCCAATTTCCTC
Cgi-58	TGGGGTTTTCCTGAGCGAC	GGTTAAAGGGAGTCAATGCTGC
Plin1	TCACCTACAGCACCCTTGTG	GGTGGAGGGAGTTTACACGA
Adrb1	GCTCTGGACTTCGGTAGATGTG	CGTCAGCAAACTCTGGTAGCGA
Adrb2	GAGCGACTACAAACCGTCACCA	TGGAAGTCCAGAACTCGCACCA
Adrb3	AGGCACAGGAATGCCACTCCAA	GCTTAGCCACAACGAACACTCG
IL6	AGCCAGAGTCCTTCAGA	GGTCCTTAGCCACTCCT

IL10	CTGGACAACATACTGCTAACCG	CTGGACAACATACTGCTAACCG
Cpt1a	CTGATGACGGCTATGGTGTTT	GTGAGGCCAAACAAGGTGATA
Mcp1	ACTGAAGCCAGCTCTCTCTTCCTC	TTCCTTCTTGGGGTCAGCACAGAC
Cpt1b	TCTGCATGTTTGACCCAAAA	TTGCTGGAGATGTGGAAGAA
Ppara	CCTGAACATCGAGTGTCGAATA	TCTTCTTCTGAATCTTGCAGCT
Tnfα	CATCTTCTCAAAATTCGAGTGACAA	TGGGAGTAGACAAGGTACAACCC
Pgc1a	TGGACGGAAGCAATTTTTCA	TTACCTGCGCAAGCTTCTCT
Ehhadh	TGTGGGTTGGAAAGTTCGCAAAGG	AATCGCCCAGCTTCACAGAGCATA
Vlcad	5-CTGAAGAATCCTTTTGGAAAC-3	CTTTCTTGTGTTTCACCAGCT
Mcad	ACCCTGTGGAGAAGCTGATG	AGCAACAGTGCTTGGAGCTT
Ucp1	ATACTGGCAGATGACGTCCC	GTACATGGACATCGCACAGC
18S (human)	CGGCGACGACCCATTCGAAC	GAATCGAACCCTGATTCCCCGTC
METTL14 (human)	GTAGCACAGACGGGGACTTC	TTGGTCCAACTGTGAGCCAG
ATGL (human)	CCACTTCAACTCCAAGGACGA	GGCAGGTTGTCTGAAATGCC
CGI-58 (human)	ACAGACCTGTCTATGCTTTTGAC	AGGGCACATCTCCACTCTTCA
ADRB2 (human)	GCCTGTGCTGATCTGGTCAT	AATGGAAGTCCAAAACTCGCA
ADRB3 (human)	GACCAACGTGTTCGTGACTTC	GCACAGGGTTTCGATGCTG

Table S2. Primer list
